# A heme•DNAzyme activated by hydrogen peroxide catalytically oxidizes thioethers by direct oxygen atom transfer rather than by a Compound I-like intermediate

**DOI:** 10.1093/nar/gkab007

**Published:** 2021-01-21

**Authors:** Nisreen M Shumayrikh, Jeffrey J Warren, Andrew J Bennet, Dipankar Sen

**Affiliations:** Department of Chemistry, Simon Fraser University, Burnaby, British Columbia V5A 1S6, Canada; Department of Chemistry, Simon Fraser University, Burnaby, British Columbia V5A 1S6, Canada; Department of Chemistry, Simon Fraser University, Burnaby, British Columbia V5A 1S6, Canada; Department of Chemistry, Simon Fraser University, Burnaby, British Columbia V5A 1S6, Canada; Department of Molecular Biology & Biochemistry, Simon Fraser University, Burnaby, British Columbia V5A 1S6, Canada

## Abstract

Hemin [Fe(III)-protoporphyrin IX] is known to bind tightly to single-stranded DNA and RNA molecules that fold into G-quadruplexes (GQ). Such complexes are strongly activated for oxidative catalysis. These heme•DNAzymes and ribozymes have found broad utility in bioanalytical and medicinal chemistry and have also been shown to occur within living cells. However, how a GQ is able to activate hemin is poorly understood. Herein, we report fast kinetic measurements (using stopped-flow UV–vis spectrophotometry) to identify the H_2_O_2_-generated activated heme species within a heme•DNAzyme that is active for the oxidation of a thioether substrate, dibenzothiophene (DBT). Singular value decomposition and global fitting analysis was used to analyze the kinetic data, with the results being consistent with the heme•DNAzyme's DBT oxidation being catalyzed by the initial Fe(III)heme–H_2_O_2_ complex. Such a complex has been predicted computationally to be a powerful oxidant for thioether substrates. In the heme•DNAzyme, the DNA GQ enhances both the kinetics of formation of the active intermediate as well as the oxidation step of DBT by the active intermediate. We show, using both stopped flow spectrophotometry and EPR measurements, that a classic Compound I is not observable during the catalytic cycle for thioether sulfoxidation.

## INTRODUCTION

**Figure 1.**
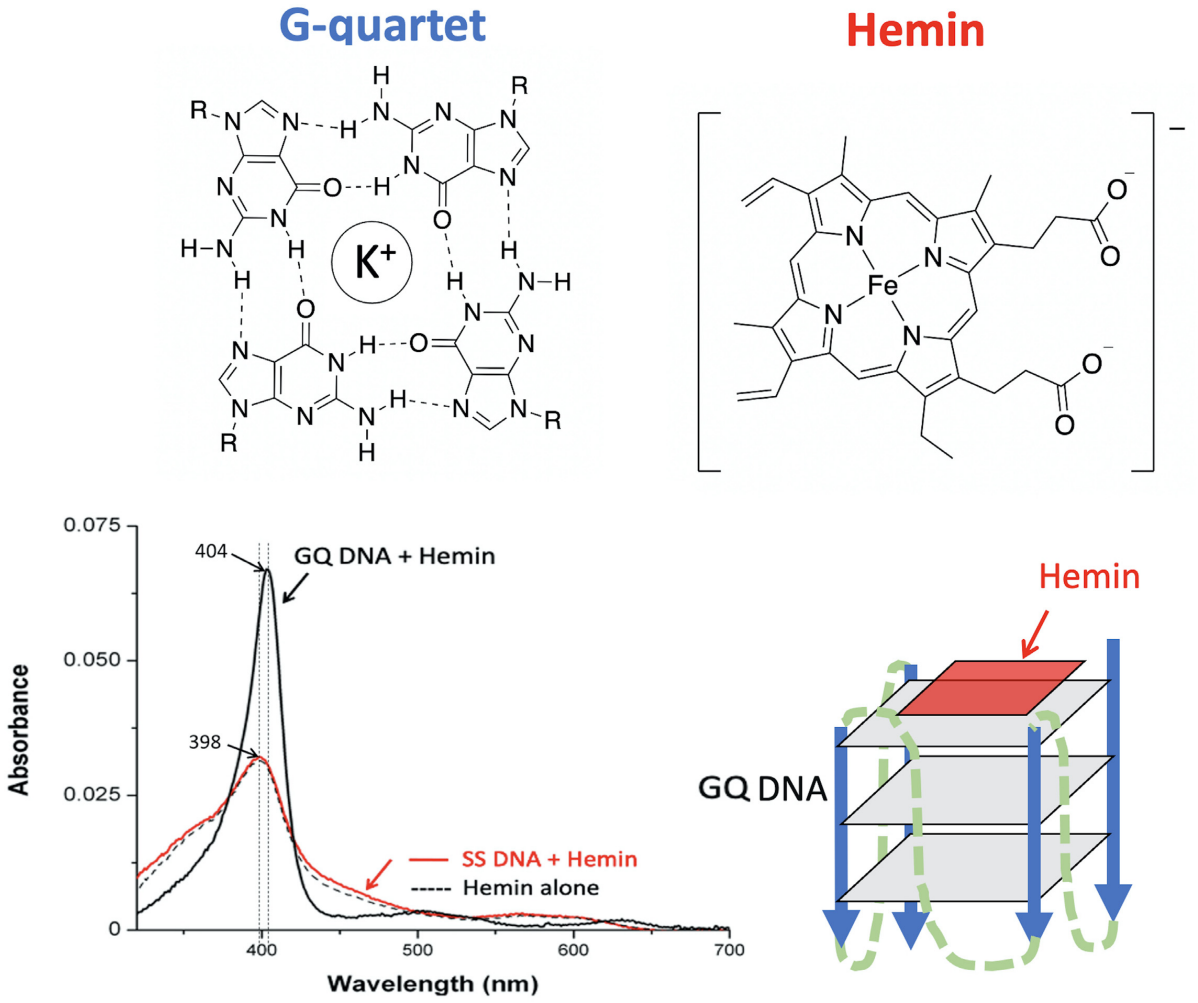
Structures of a guanine base-quartet (G-quartet) and of hemin. *Bottom left*, UV–vis spectra of hemin alone, hemin in the presence of excess non-interacting DNA (SS), and in the presence of a GQ folded DNA. *Bottom right*, a schematic of the end stacking of hemin on an intramolecularly folded GQ DNA.

Guanine-rich RNAs and DNAs that can fold into guanine quadruplexes (GQs) are known to form complexes with hemin [Fe(III)-protoporphyrin IX] (Figure 1). The resulting complex is referred to as a heme•DNAzyme or heme•ribozyme and such complexes show robust, nucleic acid-enhanced peroxidase (1e^–^ oxidation) and peroxygenase/oxygenase (2e^–^ oxidation) reactivity. Importantly, these reactions are orders of magnitude faster than those of monomeric hemin disaggregated by surfactants (1–5). It is widely reported that heme•DNAzymes and heme•ribozymes, including sequences derived from genomes, are efficient catalysts *in vitro* and can be comparable to naturally occurring hemoproteins, particularly in oxidation of thioether substrates (1–8). The occurrence of such a nucleic acid-enhanced oxidative activity has raised questions about its likely biological relevance. Crucially, several separate lines of investigation have recently reported that such activity is indeed operational in living cells (9–12). An important ongoing question in this field, therefore, is the identity of the catalytically active heme species within heme•DNAzymes relevant to different substrate oxidations.

The nucleic acid partner of the heme-nucleic acids complex provides a unique chemical environment for the bound hemin, whose UV-vis spectrum closely resembles those of oxidative hemoproteins, such as metmyoglobin and horseradish peroxidase (HRP) (1). UV–vis (1) and EPR (3) studies of the heme•DNAzyme have shown that the heme iron is hexacoordinated at pH 7–8 and is present in a high-spin state. Recent ^1^H NMR experiments by Yamamoto *et al.* has established that the distal axial coordination site is occupied by a water molecule, which is positioned in the central cavity of the most proximal G-quartet (6–8). Such water coordination, compared to the commonly found imidazole (in peroxidases) or thiolate coordination (in cytochromes P450) is central to addressing the question of the nature of the activated heme species in H_2_O_2_ activated heme•DNAzymes.

Reactivity of heme•DNAzymes is diverse and includes 1e^–^ and 2e^–^ oxidation reactions (e.g. thioether oxidation, alkene epoxidation, and oxidation of indole) (4). These reactions closely resemble reactions carried out by heme peroxidases and oxygenases. In those hemoproteins, an activated iron species called ‘Compound I’ is the initial active intermediate. Compound I is typically formulated as O = Fe^IV^{porphyrin•+} (13–15). In some hemoproteins, however, such as cytochrome c peroxidase (CcP), the initial species observable is not a classic Compound I. In that case, the radical cation is delocalized on a proximal tryptophan rather than on the porphyrin itself (16–18). Such an activated compound was originally known as Compound ES or recently as Compound I’ to distinguish it from the classic Compound I.

**Figure 2.**
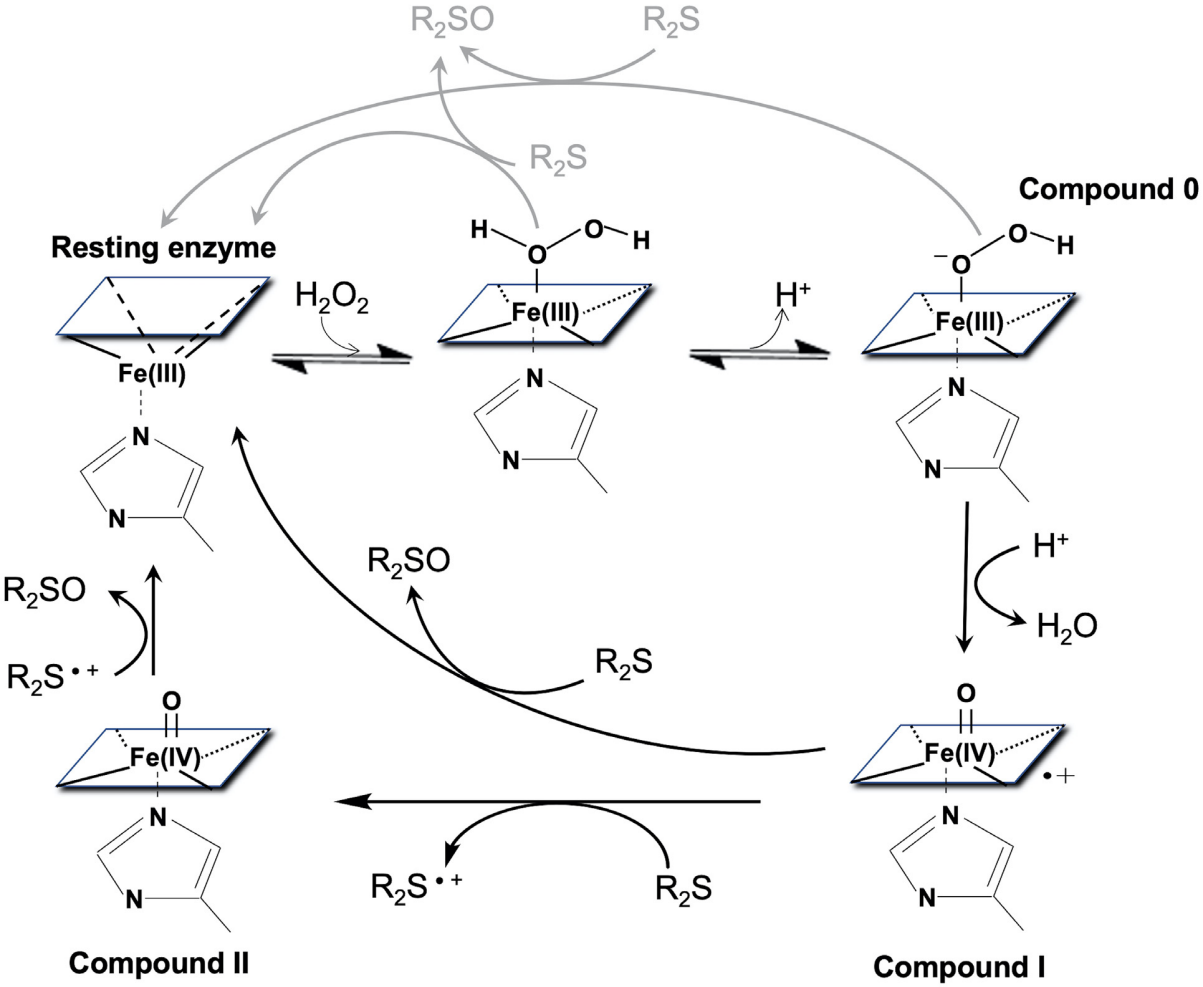
A generic peroxidase catalytic cycle for the oxidation of thioether substrates (R_2_S) to Sulfoxide products (R_2_SO).

The above studies inspired our thinking about the reactive intermediates in heme•DNAzymes. The well established peroxidase cycle for the oxidation of thioether substrates (R_2_S) is shown in Figure 2. Here, H_2_O_2_ binds heme and is then deprotonated to a ferric hydroperoxy species (Compound 0). Protonation of the distal O and loss of water generates Compound I, which can oxidize R_2_S substrates. The O-atom transfer reaction can either proceed via two sequential 1-electron oxidation reactions through a Compound II (O = Fe^IV^porphyrin) intermediate, or by direct O-transfer. The latter mechanism is favored for horseradish peroxidase and CcP, based on isotope labeling experiments with H_2_^18^O_2_ (19,20). However, Compound II has been detected during the thioether oxidation cycle of HRP (21), so sequential 1e^–^ (also called rebound) mechanism cannot be ruled out in all cases. With cytochrome P450 monooxygenases the rebound mechanism is commonly discussed, but two other species, the Fe(III)–H_2_O_2_ and ferric hydroperoxo complexes (Compound 0) have also been proposed to be capable of catalyzing thioether oxidation reactions (15,22–24).

Heme•DNAzymes do have some key distinguishing features with respect to heme proteins. Thus, Wang *et al.* (28) and Shangguan *et al.* (29) demonstrated that the peroxidase activity of heme•DNAzymes was dependent on H_2_O_2_ but not on the ABTS substrate concentration (28). Heme•DNAzymes show a broader substrate range than HRP, likely due to the former's exposed catalytic center (2,30). One study in particular found that the limited catalytic lifetime of heme•DNAzymes was due to eventual degradation of the complexed heme rather than major destruction of the GQ DNA (29).

Time-resolved UV–vis spectroscopy is an excellent tool for studying the identity of activated heme species. Compounds I and II in heme proteins are distinguishable on the basis of their spectroscopic features. Typically, Compound I from HRP is characterized by its diminished Soret absorption intensity relative to the Soret bands of other heme states (25); by contrast, because the aromaticity of its porphyrin ring is maintained, the absorption for CcP Compound I’ is undiminished relative to that of the resting enzyme and resembles that of Compound II (26). The visible region of the UV-vis spectrum, too, provides valuable information about the spin state and oxidation state of the heme. (27).

The UV–vis spectroscopic characteristics of resting heme•DNAzymes are well-known (1). The Soret band exhibits significant hyperchromicity and a red shift of 5–6 nm relative to that of uncomplexed, monomeric hemin (Figure 1). The visible spectrum of heme•DNAzymes resembles those of metmyoglobin and HRP. An early study that probed the mechanism of oxidation by heme•DNAzymes looked at changes in the UV–vis absorption bands upon treatment of a heme•DNAzyme with H_2_O_2_ (in the absence or the presence of the reducing substrate hydroquinone (H_2_Q)) (3). That study found that the Soret band amplitude of the heme•DNAzyme remained substantially unchanged in the presence of H_2_Q, whereas it progressively decayed in absence of H_2_Q, exhibiting some features consistent with a Compound I-like species (3). EPR experiments on the H_2_O_2_-treated samples showed the formation of an organic radical with a simple singlet EPR signal; this intermediate was assigned as a carbon centered radical, most likely localized on a guanine base (3).

The use of UV–vis spectroscopy to study activated heme•DNAzyme intermediates has provided limited information to date, owing to a lack of temporal resolution. Herein, we use stopped-flow diode array UV-vis spectroscopy to gather time-dependent spectra (300–750 nm) to investigate the identity or identities of the activated heme species formed following treatment of a heme•DNAzyme with H_2_O_2_—both in the presence and absence of a thioether substrate, dibenzothiophene (DBT). Our goal was to probe whether the oxidation of *thioethers* by heme•DNAzymes operates *via* a classic Compound I or via one of the other mechanisms described above.

## MATERIALS AND METHODS

DNA oligonucleotide CatG4 (‘G4’); [5′-TGG GTA GGG CGG GTT GGG AAA-3′], and single stranded DNA (‘SS’) [5′-AAT ACG ACT CAC TAT ACT-3′] used for this study were purchased from the University Core DNA Services (University of Calgary). The oligonucleotides were size-purified by gel purification followed by standard desalting methods. Purified DNA was dissolved in TE buffer (10 mM Tris, pH 7.5, 0.1 mM EDTA) and frozen at –20°C until needed. Folding of the oligonucleotide to a GQ was achieved by incubation of the oligonucleotide in Reaction Buffer (40 mM HEPES–NH_4_OH, pH 8.0, 20 mM KCl, 25% methanol, 1% DMF and 0.05% Triton X-100). Hemin was purchased from Frontier Scientific (Logan, UT, USA). A 10 mM stock of hemin was prepared in DMF and stored in dark at –20°C and was diluted and used as needed. Dibenzothiophene (DBT) and dibenzothiophene sulfoxide (DBTO) were purchased from Santa Cruz Biotechnology Inc., Dallas, TX, USA. A stock of 10 mM DBT was prepared in methanol and stored at 4°C. ^18^O-Hydrogen peroxide was purchased from Icon Isotopes, Dexter, MI. All other chemicals were from Sigma-Aldrich.

### Stopped-flow kinetics experiments

All experiments were performed in a SX20 stopped-flow spectrophotometer (Applied Photophysics), using a 2 mm path-length cell and a standard 20 μL cell volume. The dead time of the stopped-flow using this cell was determined to be 1 ms. Detection of time-resolved spectra at multiple wavelengths was achieved with an Applied Photophysics Photodiode Array Accessory (PDA), capable of an integration speed of 1.4 ms per scan. Time-resolved spectra were recorded in the 180–740 nm spectral range, and 1000 spectra were collected within periods of 10, 60 and 200 s. The sample handling unit of the instrument was equipped with a water bath to provide temperature control of the stopped-flow experiments. A circulator unit was used to pump thermostatic fluid into the water bath housing surrounding the drive syringes, drive valves and flow tubing. The thermostatic fluid also filled the cell block, ensuring temperature control of the entire flow circuit. Experiments were performed at 21.0 ± 1 and 4.0 ± 1°C.

For single mixing experiments, two DNA solutions (of the G4 oligonucleotide, and of the single-stranded control oligonucleotide SS), as well as a solution of the heme alone control were prepared in 2× reaction buffer (80 mM HEPES–NH_4_OH, pH 8.0, 40 mM KCl, 50% methanol, 2% DMF, 0.1% Triton X-100). The two DNA solutions were incubated at 20°C for 10 min for appropriate DNA folding. Hemin was then added to both solutions followed by a further 10 min incubation at 20°C to insure full hemin–DNA interaction. For the solutions containing substrate, DBT was added last. For the ‘heme alone’ solution, an equivalent volume of TE buffer was added instead of DNA. Hydrogen peroxide solutions were diluted freshly from the stock into reaction buffer. Solution A, containing heme, DNA (present or absent), and DBT (present or absent) was connected to stopped-flow syringe A, and Solution B, containing the hydrogen peroxide, was connected to syringe B. Solution A and Solution B were mixed in a 1:1 mixing ratio, with the resulting final concentrations in the optical observation cell after mixing being: 7 μM heme, 50 μM DNA, 0/100 μM DBT, and 0.007/0.5/100 mM hydrogen peroxide in the reaction buffer (40 mM HEPES–NH_4_OH, pH 8.0, 20 mM KCl, 1% DMF, 25% methanol, 0.05% Triton X-100).

### Software and kinetic data analysis

Experimental traces recorded in the 300–740 nm spectral range were processed using the Pro-KIV Global Analysis Software (Applied Photophysics). Singular value decomposition (SVD) was performed, by which the data matrix was decomposed into three matrices with the form of: Y = U • S • V^T^

Each column of the U matrix represents the time evolution of the reaction. U is a matrix of column vectors where each column is an eigenvector in the time domain. V^T^ is a matrix of column vectors where each column is an eigenvector in the wavelength domain. S is a diagonal matrix with the singular values S. Each element of S represents the contribution of the corresponding basis spectra to the observed data.

The SVD output was analyzed by global optimization of the provided reaction parameters using the Marquardt–Levenberg algorithm. Various models for the reaction were input into the analysis equations window, as successive steps. Compilation generates the rate parameters associated with each step of the model. Spectra for the starting material (the ferric DNAzyme), DBT, and DBTO were collected under the same experimental conditions, averaged and saved as single spectra, then used by the software. Hydrogen peroxide was included in the reaction scheme but not incorporated into the regression analysis as it is assigned to be a colorless species.

In order to run the analysis, the concentrations of each of the starting materials (heme/G4-DNAzyme, H_2_O_2_ and DBT) were entered, with an initial estimate given for each rate constant. The rate of DBTO formation (*k*_2_) was determined previously by fitting the change in absorbance over time at 334 nm using GraphPad Prism. These values were used as the ‘initial guesses’ for the rate constants by the analysis process. Upon completion, rate constants, component spectra, and their corresponding concentration profiles could be displayed. The calculated rate constants obtained from the software analysis were reported as mean values obtained from three replicate experiments, along with their standard deviation errors. The software also calculates the wavelength residual plots, which illustrate the global accuracy of the selected model. Supplementary Figure S1 sets out the order of steps by which the analysis process is accomplished.

A number of models and values of rate parameters were tested in order to achieve final calculated spectra and residual plots consistent with the presence of no additional species (i.e. final fits showed no systematic deviations). Furthermore, to test the robustness of each hypothesized model, individual reaction steps were removed systematically from the scheme, followed by re-analysis and inspection of residual plots. Further details of the methods used for the kinetic data analysis are given in the Supporting Information.

### Sulfoxidation of DBT to DBTO using H_2_^18^O_2_

GQ-forming oligonucleotide (‘G4’) from a stock solution was added to reaction buffer (40 mM HEPES–NH_4_OH, pH 8.0, 20 mM KCl, 1% DMF, 25% methanol, 0.05% Triton X-100) to a final concentration of 50 μM and rested for 5 min to ensure folding. Heme was then added to a final concentration of 7 μM, followed by a 10 min incubation at 20°C to ensure binding. Substrate (DBT) was then added, to a final concentration of 200 μM. The oxidative reaction was initiated by adding either H_2_^16^O_2_ or H_2_^18^O_2_ (90 atom%, Icon Isotopes, Summit, NJ, USA) to a final concentration of 1 mM. For a negative control, an equivalent volume of ddH_2_O was added instead of hydrogen peroxide. The reaction was allowed to proceed for 30 min; an equal volume of dichloromethane was then added to extract the DBT and any DBTO product. The organic solvent was removed by overnight evacuation, and the solid residue dissolved in 10 μL methanol for analysis by ESI-MS (Supporting Information). The level of ^18^O incorporation into the DBTO product was calculated by the method of Brauman (32).

### EPR experiments

EPR measurements were performed at X-band (9.3–9.4 GHz) using a Bruker EMXplus spectrometer with a PremiumX microwave bridge and HS resonator. Spectra were recorded for all samples at 100 K. Concentrations of hemin, DNA, and H_2_O_2_ were identical to those used for UV–vis kinetics experiments (7 μM heme, 50 μM DNA and 0.5 mM hydrogen peroxide in the reaction buffer: 40 mM HEPES–NH_4_OH, pH 8.0, 20 mM KCl, 1% DMF, 25% methanol, 0.05% Triton X-100). For samples with added H_2_O_2_, the mixtures were prepared directly in the EPR tube and frozen in liquid nitrogen within 15 s of mixing.

## RESULTS

### General considerations

**Scheme 1.**
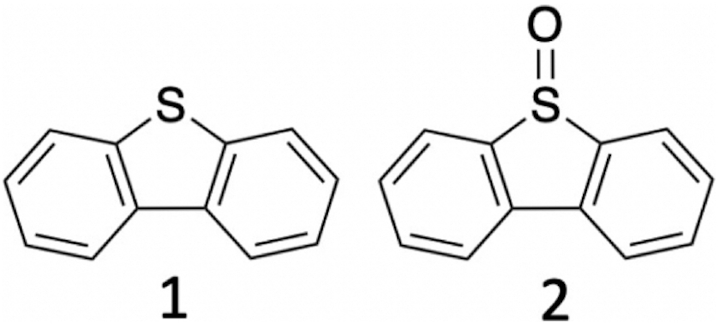
(1) Dibenzothiophene (DBT); (2) dibenzothiophene oxide (DBTO).

DBT was chosen because it is readily oxidized to dibenzothiophene sulfoxide (DBTO) by the heme•DNAzyme (Scheme 1). Significantly, the optical spectra of DBT and DBTO are distinct, which allows for characterization of oxidation kinetics. Furthermore, neither spectrum overlaps with those of resting heme or of the different activated heme species. DBT has a low water solubility (20 μM); however, we have shown that heme•DNAzymes work exceptionally well in aqueous buffers containing up to 40% MeOH (31). Here, 25% methanol was added to reaction buffers. Following data collection, fitting and global analysis of the multivariate data sets were carried out to define both time-dependent spectral changes and the UV–vis spectra of putative reaction intermediate(s).

To ensure quantitative heme binding by the folded G-quadruplex (G4), we used DNA concentrations in a7-fold excess with respect to hemin. Under these conditions, 99% of the hemin is DNA-bound,based on the dissociation constant (*K*_d_) of the complex (1,33). We used a sufficient concentration of hemin (7 μM) to enable observation of reactive intermediates. Of note, the 25% methanol used to enhance the solubility of DBT is known not to interfere with the structure of G-quadruplexes (30).

### Optimization of DBT oxidation reaction conditions

The effect of varying H_2_O_2_ concentration on the production of activated heme•DNAzyme intermediates was first investigated. Initial experiments probed the possibility of generating H_2_O_2_-activated intermediates of the heme•DNAzyme under conditions of excess peroxide in the absence of substrate. In the literature, detection of high-valent intermediates (Compounds I and I’) from the native and mutant metmyoglobins has been reported in the absence of reducing substrate and under conditions of high H_2_O_2_ concentration (up to 50–100 mM, relative to 5 μM enzyme) (34,35). With regard to heme•DNAzymes, an earlier comparison of their absorbance parameters (λ_max_ and ϵ_M_; Soret maxima, and D and E charge transfer bands) with those of different hemoprotein complexes had suggested significant similarities with metmyoglobin (1). Conditions similar to those used for the study of the active intermediates of metmyoglobin were used. The observed spectral changes at high H_2_O_2_ concentrations suggested extensive and rapid heme degradation in the heme•DNAzyme, as indicated by the permanent decline and eventual loss of the Soret spectral peak (Supplementary Figure S2). Such heme degradation has been also observed for hemoproteins at high oxidant concentrations (36–42). A stoichiometric (7 μM) concentration of H_2_O_2_ was then investigated. Stoichiometric amounts of H_2_O_2_ have been used for the study of HRP, allowing for the detection of HRP’s Compound I by stopped flow UV–vis (43,44). However, the combination of 7 μM heme•DNAzyme and 7 μM hydrogen peroxide showed no appreciable change in the heme's Soret absorption over 10 s) (Supplementary Figure S3), a finding consistent with previous reports that at least 40–200 equivalents of peroxide are required to observe significant changes in the optical spectrum of heme•DNAzymes over a 3–5 min timescale (3,41). We found intermediate H_2_O_2_ concentrations (0.5–1.0 mM) to be optimal, and this concentration range was used for most of the experiments reported herein.

Next, the reactions with DBT test substrate were explored. Specifically, to check if the presence of DBT in the methanol-containing aqueous reaction solution would change the heme•DNAzyme absorption, the UV–vis spectra of the DNA and hemin in reaction buffer (in both the presence and absence of DBT) were measured in the 300–740 nm range (Supplementary Figure S4). No change in the heme•DNAzyme's spectrum was observed. We found that 0.5 mM hydrogen peroxide was sufficient to induce a spectral change of the heme/G4 complex (7 μM) in ∼45 s and, at the same time convert DBT to DBTO (in >80% yield).

### Single mixing experiments in the presence and absence of DBT

**Figure 3.**
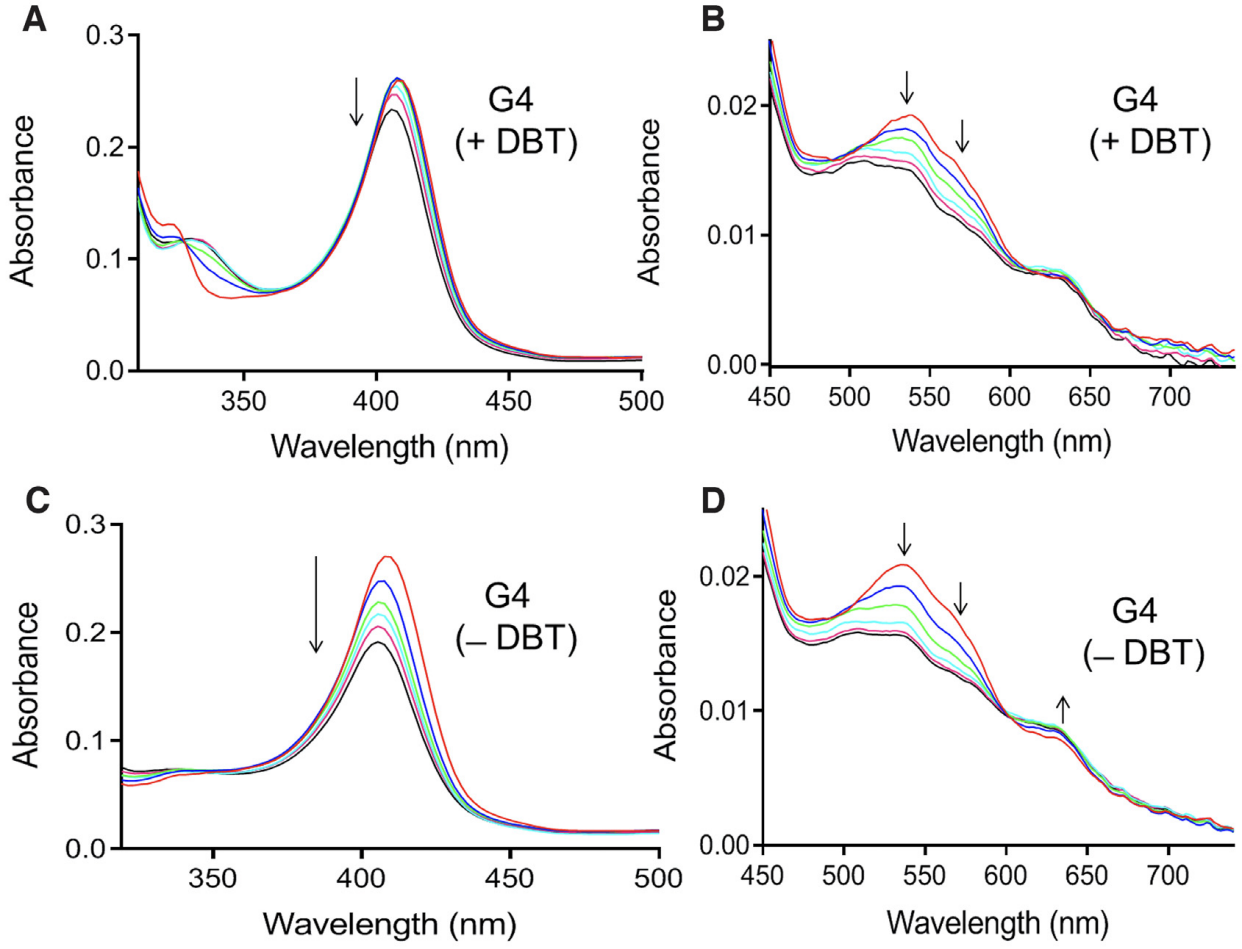
Time-dependent changes in the spectra, over the first 200 s of reaction, of the G4–hemin complex (heme•DNAzyme) in the presence (**A**, **B**) and absence (**C**, **D**) of the added DBT substrate.

**Figure 4.**
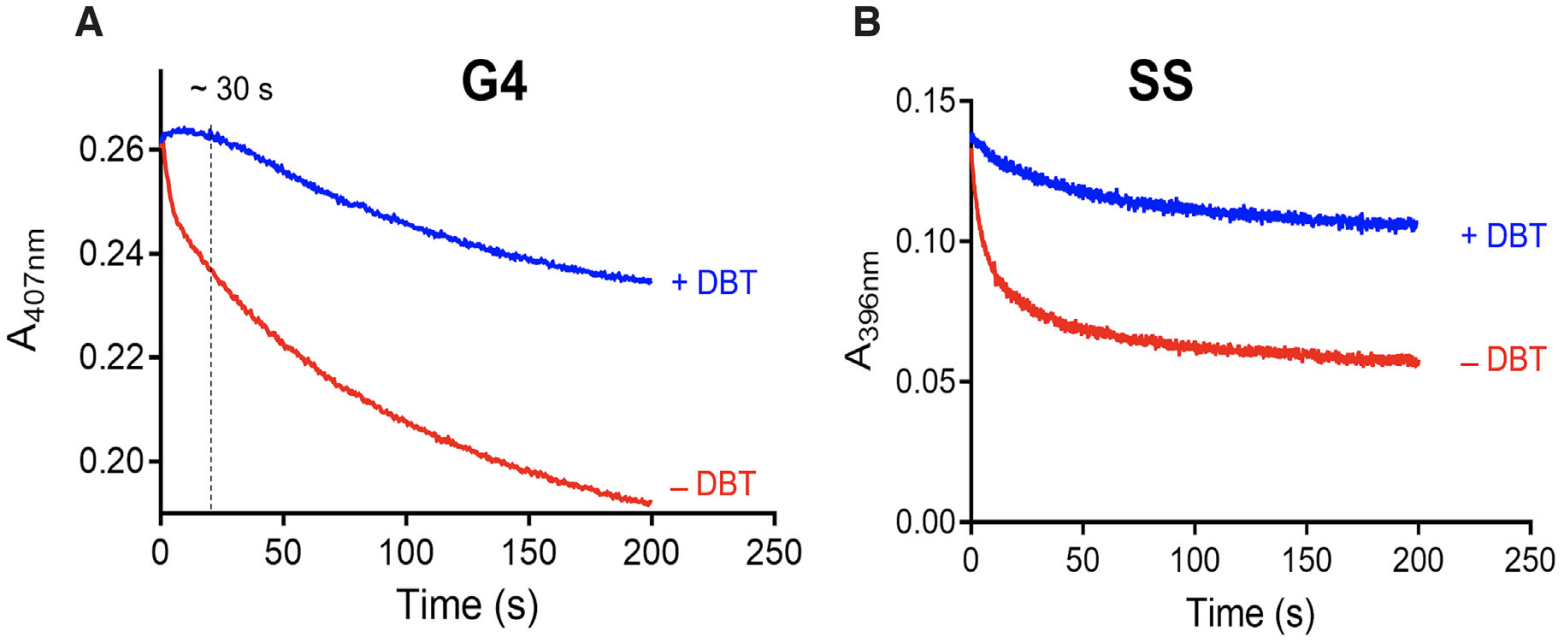
(**A**) Absorbance at the Soret maximum (407 nm) for the G4–hemin complex (heme•DNAzyme) plotted as a function of time, in the presence and absence of the DBT substrate. (**B**) Absorbance of the hemin Soret maximum (396 nm), in the presence of SS DNA, plotted against time, again in the presence and absence of the DBT substrate. Time = 0 s traces are shown in red and time = 200 s in black.

Single mixing stopped-flow experiments were applied both to a catalyzed reaction, i.e. in the presence of the heme•DNAzyme, and to an uncatalyzed reactions, with the same concentrations of either hemin alone or hemin and a non-G-quadruplex-forming oligonucleotide (‘SS’). Both uncatalyzed reaction negative controls gave rise to similar spectroscopic data showing barely any catalysis (Supplementary Figure S5). For the subsequent data analysis, we analyzed the ‘SS’ control. Time-dependent spectral changes upon mixing the DNA (G4 or SS) with 0.5 mM H_2_O_2_ at 21°C were recorded from 300 to 742 nm over 200 s in the presence or absence of DBT. Figure 3 shows data for the catalyzed reactions of the heme•DNAzyme (G4) with and without DBT. Data for heme + SS DNA are shown in the Supporting Information (Supplementary Figure S6). Corresponding kinetics traces for both the G4 and SS reactions are shown in Figure 4.

Reactions of mixtures of H_2_O_2_ and heme•DNAzyme show some noteworthy spectral changes. First, in the presence of DBT, the Soret band was stable for ∼25–30 s (Figure 3A), followed by a slow decay in amplitude. In the absence of DBT, only progressive loss of Soret intensity was observed over the same timescale (Figure 3C). There is also a slight increase at 633 nm and a decrease at 570 and 530 nm intensities (Figure 3D).

**Figure 5.**
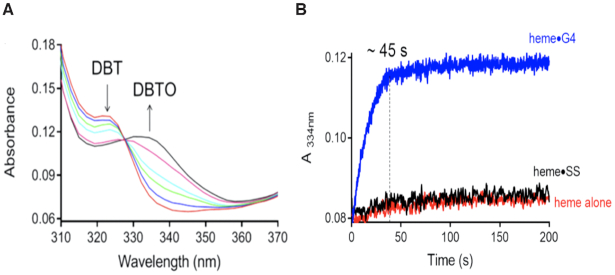
(**A**) Time-dependence of spectral changes that are associated with the conversion of DBT to DBTO. Time = 0 s traces are shown in red and time = 200 s in black. (**B**) Plots of the change in absorbance at 334 nm over 200 s time in the catalyzed reaction of heme•G4 (blue trace) and the uncatalyzed reactions of heme•SS (black trace) and heme alone (red trace).

Analysis of the plots of Soret absorbance (407 nm) versus time also provide insight into the reaction progress. As noted above, the presence of DBT gives rise to a Soret intensity that remains stable for ∼30 s. This is consistent with a reaction of a sulfoxidation-competent heme species with DBT to produce DBTO—a conclusion supported by the time profile of DBTO production. Figure 5 shows that conversion of DBT to DBTO by the heme•DNAzyme is complete in 45 s. In the absence of DBT, different behavior is observed; a competing process, clearly visible in Figure 4A, shows the decay of the Soret band of the heme•DNAzyme. A sharp decline in the intensity of the Soret band occurs within ∼2 s (Figures 3C and 4A), likely signifying the time required for the formation of the active intermediate. This is followed by continuous decay of the Soret peak, consistent with heme degradation.

The time-dependent spectra for the uncatalyzed reactions (i.e. in the presence of SS DNA) are shown in Supplementary Figure S6. The difference in the patterns of Soret absorbance decline in the presence and absence of DBT even in these DNA-uncatalyzed (Figure 4B, *right*) reactions suggests that the nature of the active complexes is different. DBT offers minimal protection against heme degradation in reactions of heme + SS, suggesting that the intermediate is not competent for sulfoxidation.

**Figure 6.**
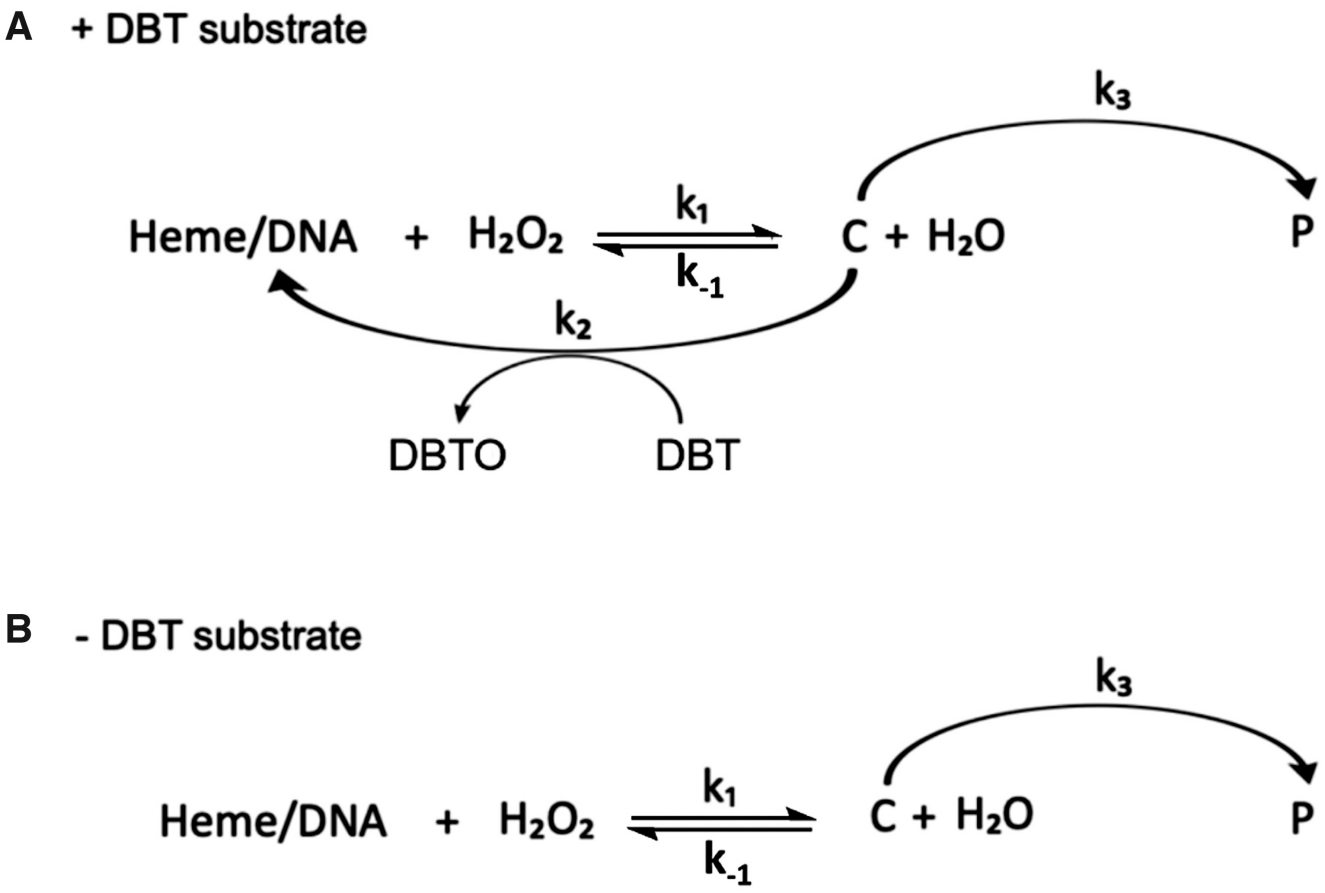
Proposed reaction schemes in the presence (**A**) and absence (**B**) of the substrate DBT. C is the activated species; P is the degraded product. *k*_1_ and *k*_2_ are second-order rate constants, while *k*_–1_ and *k*_3_ are first-order rate constants. Note that ‘DNA’ in the schemes, above, refers to either the G4 forming oligonucleotide or SS oligonucleotide.

**Figure 7.**
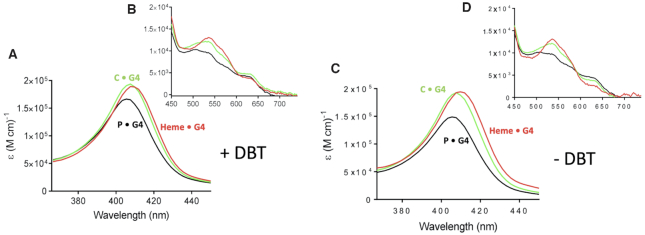
Deconvolved spectra of hemin complexed with G4 DNA (heme•DNAzyme) in the presence of the DBT substrate (**A**, **B**); and in the absence of the DBT substrate (**C**, **D**).

To deconvolve the time-dependence of spectral changes associated with the appearance and disappearance of all species involved in the reaction time course, the collected data sets shown in Figure 3 were first reduced by singular value decomposition (SVD), as described in the Methods section and then processed by a global fitting routine. Figure 6 displays our mechanistic hypothesis for the reaction scheme, which we tested for the data shown in Figure 3 and Supplementary Figure S6. In this model, reaction of heme•DNA with H_2_O_2_ reversibly generates an active intermediate, ‘C’. When DBT is present, two competing reactions take place. First, C can react with DBT to give DBTO, with a concomitant regeneration of the resting heme•DNAzyme. When H_2_^18^O_2_ is used as the oxidant, the yield of DBT^18^O is 97.8%, in support of the model where the transferring [O] originates from H_2_O_2_ (Supplementary Figure S7). In a competing reaction, C proceeds to a degradation product, ‘P’ (Figure 6). In the absence of DBT substrate (Figure 6) C converts exclusively to P. Using this model, a deconvolved spectrum for each species (heme•G4, C and P) can be obtained from the global analysis. Individual spectra are shown in Figure 7 (for heme•DNAzyme) and, in Supplementary Figure S8 (for SS DNA).

In Figure 7 and Supplementary Figure S8, the deconvolved spectra of heme•G4 and hemin + SS DNA are shown in red, those of species C•SS and C•G4 in green, and of the initial degradation product, P•SS, and P•G4 in black. Tight residuals plots showing no systematic deviations are shown in Supplementary Figure S9a and e, supporting the validity of the reaction model shown in Figure 6. As a test of the robustness of this model, individual reaction steps were systematically removed from the model, followed by re-analysis and inspection of residual plots. Supplementary Figure S9b–d, f, g shows that these alternative reaction models are characterized by large and systematic deviations in their residual plots, consistent with the presence of exactly three species in the kinetic model.

Notably, the Soret band of the activated species, C•G4 (green, Figure 7) is strikingly similar to that of the resting heme•DNAzyme (red); the latter shows an undiminished amplitude and a slight (∼2 nm) blue-shift of its maximum. Also, less intense peaks for both species are seen at 530, 582 and 633 nm, with a shoulder at ∼506 nm. The species P•G4 has a Soret band at 406 nm and visible peaks at 500, 533, 580 and 631 nm.

### The kinetics of DBT sulfoxidation

The second order rate constants, *k*_1_ and *k*_2_, and the first order rate constants, *k*_–1_ and *k*_3_, obtained from the global fit, are summarized in Table 1. The values of *k*_1_ and *k*_–1_ in the catalyzed reaction, describing the formation and the dissociation of the activated species, are independent of DBT, and both are 1 to 2 orders of magnitude higher than the *k*_1_ and *k*_-1_ values for the analogous uncatalyzed reaction. The rate constant *k*_3_ describes the heme inactivation reaction. Again, both the heme•DNAzyme-catalyzed and the heme•SS (uncatalyzed) reaction yield comparable values, regardless of the presence of DBT. Most significantly, the rate constant *k*_2_, which describes the DBT oxidation step, is two orders of magnitude larger for the heme•DNAzyme-catalyzed reaction than for the uncatalyzed reaction. These differences in *k*_1_ and *k*_2_ for reactions of the heme•DNAzyme and the uncomplexed hemin (+SS) highlight two key roles of the DNA GQ: (a) in the generation of the activated heme species; and (b) in the formal oxidation step of DBT to DBTO.

**Table 1.** Rate constants describing the oxidation of DBT to DBTO

Rate constants	Catalyzed reactions		Uncatalyzed reactions	
	+ DBT	− DBT	+ DBT	− DBT
*k*_1_ (M^–1^s^–1^)	(1.6 ± 0.2) × 10^3^	(1.2 ± 0.3) × 10^3^	(1.7 ± 0.3) × 10^2^	(1.9 ± 0.5) × 10^2^
*k*_-1_ (s^–1^)	(13.5 ± 0.2) × 10^3^	(12.2 ± 1.6) × 10^3^	(3.5 ± 0.5) × 10^2^	(3.6 ± 0.4) × 10^2^
*k*_2_ (M^–1^s^–1^)	(5.4 ± 1.0) × 10^2^	N/A	(0.1 ± 0.05) × 10^2^	N/A
*k*_3_ (s^–1^)	(1.05 ± 0.02) × 10^−2^	(1.35 ± 0.02) × 10^−2^	(2.35 ± 0.04) × 10^−2^	(1.50 ± 0.03) × 10^−2^

Rate constants *k*_1_, *k*_-1_, *k*_2_ and *k*_3_ (see Figure 6) are each reported as the mean of three replicate experiment, with their standard deviation.

To see if we could capture any species other than heme•G4, C•G4 and P•G4, we carried out the stopped-flow experiment also at 4°C. We observed slower kinetics at 4°C, but no sign of any species other than the ones we observed at 21°C. The results of that experiment and the calculated rate constants are shown in Supplementary Figure S10.

### EPR experiment

**Figure 8.**
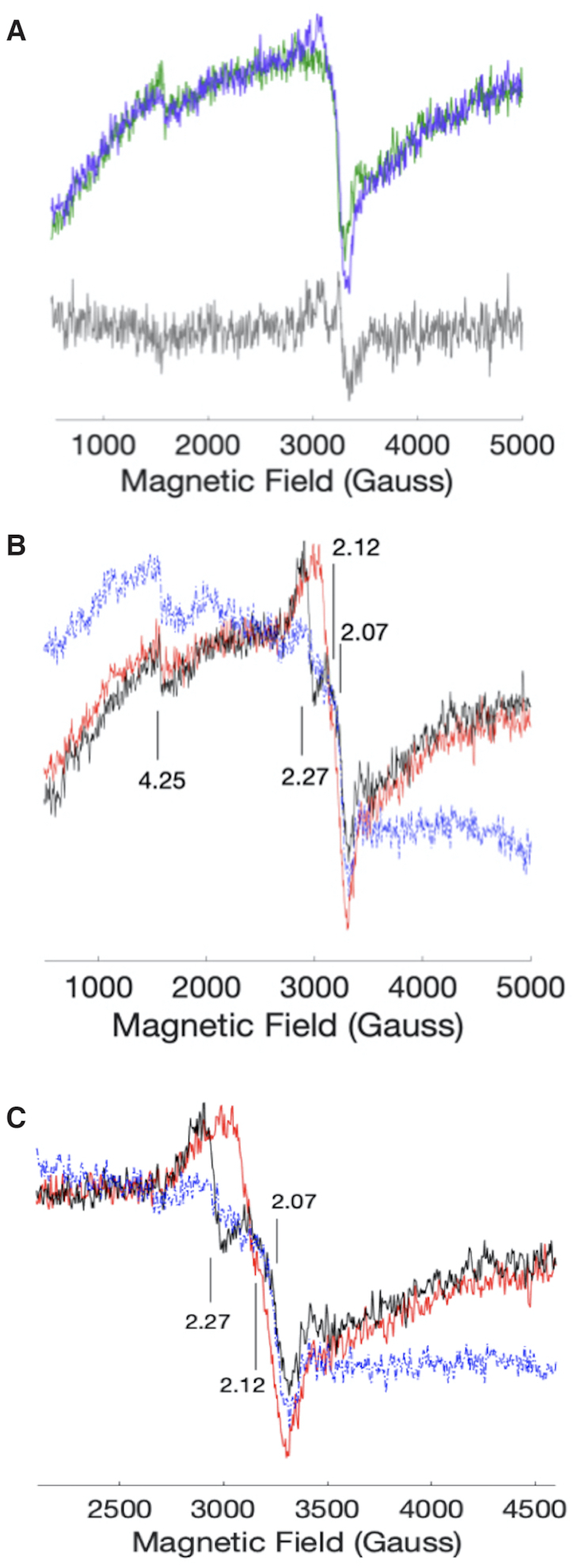
(**A**) EPR spectra of SS DNA (green) and SS DNA + H_2_O_2_ (purple). The concentrations are 50, 7 and 500 μM of DNA, heme, and H_2_O_2_, respectively. The grey trace is the difference spectrum of the two samples. (**B**) EPR spectra of G4•heme (blue); G4•heme added to a solution of H_2_O_2_ (black); and, a solution of H_2_O_2_ added to G4•heme (red). (**C**) an x-axis expanded version of panel (b).

To explore in greater depth the conclusions derived from the above stopped-flow UV–vis spectrophotometry experiments, we carried out EPR experiments, using the same reagent (DNA; heme; and H_2_O_2_) concentrations as were used in the stopped-flow UV–vis experiments. Figure 8A shows The EPR spectra of SS DNA alone (in blue), and of SS DNA + H_2_O_2_ (in red). The two spectra exhibit few differences, as can be seen from the computed difference spectrum between the two spectra (shown in black). Addition of H_2_O_2_ gives rise to a very weak signal with *g* ∼ 2.05. There are no obvious products at lower fields.

The EPR spectrum of G4•heme (Figure 8B and C) shows two features, at *g* = 2.07 and at *g* = 4.24, that are consistent with a high-spin Fe(III) heme (3). Using the pre-formed G4•heme complex, two additional EPR sample preparations were tested. In the first, a solution of H_2_O_2_ was added to the G4•heme solution in the EPR tube. In the second sample, the order of addition was reversed (G4•heme was added to a solution of H_2_O_2_). The resulting EPR spectra are shown in Figure 8B and C). In all EPR solutions, the concentrations of all constituents were the same as in the stopped-flow experiments, to allow for more straightforward comparison. In both orders of mixing, a small signal at *g* = 4.24 was present, which is consistent with a small amount of the unreacted heme starting material. Mixing of G4•heme and H_2_O_2_ gave rise to signals near *g* = 2, regardless of the order of addition (Figure 8B and C). When G4•heme was added to an H_2_O_2_ solution, signals at *g* = 2.27 and *g* = 2.07 were observed. In the reverse order of addition, a signal near *g* = 2.12 was observed. The origin of this behaviour is not known. Upon warming, samples of G4•heme + H_2_O_2_ showed a substantial loss of EPR signal, consistent with heme degradation observed by stopped-flow (Supplementary Figure S11).

The EPR spectra do not show signals consistent with Compound I-like intermediates. Such signals are very close to *g* = 2, as in cytochrome *c* peroxidase, where the formulation is a O = Fe^IV^porphyrin and a proximal tryptophan radical (45). Likewise, a *g* = 2 signal for a O = Fe^IV^porphyrin^•+^ intermediate is not observed (46). In principle, Compound I intermediates, reminiscent of those in horseradish peroxidase (46) and P450 enzymes (47), could be present at very low concentrations, and thus difficult to observe at temperatures above 4 K.

Based on the EPR and stopped-flow kinetics data, we propose the intermediacy of a unique low spin (S = $\frac{1}{2}$) six coordinate heme (48). This proposal is in accord with the computational prediction of an S = $\frac{1}{2}$ adduct of heme + H_2_O_2_ (22). Other EPR studies of S = $\frac{1}{2}$ ferric neuronal nitric oxide synthase (nNOS) show *g*-values in the same range as we observe (*g* ∼ 2.1–2.2) (49). In addition, our extracted optical spectra show features that are also observed in NOS enzymes, in particular in the shapes of the Q bands (50). Thus, both the UV–visible spectral and EPR data support the presence of a low spin complex resulting from mixing G4•heme with H_2_O_2_. Our kinetics analysis suggests that this is the active oxidant in oxygen transfer to DBT.

## DISCUSSION

**Figure 9.**
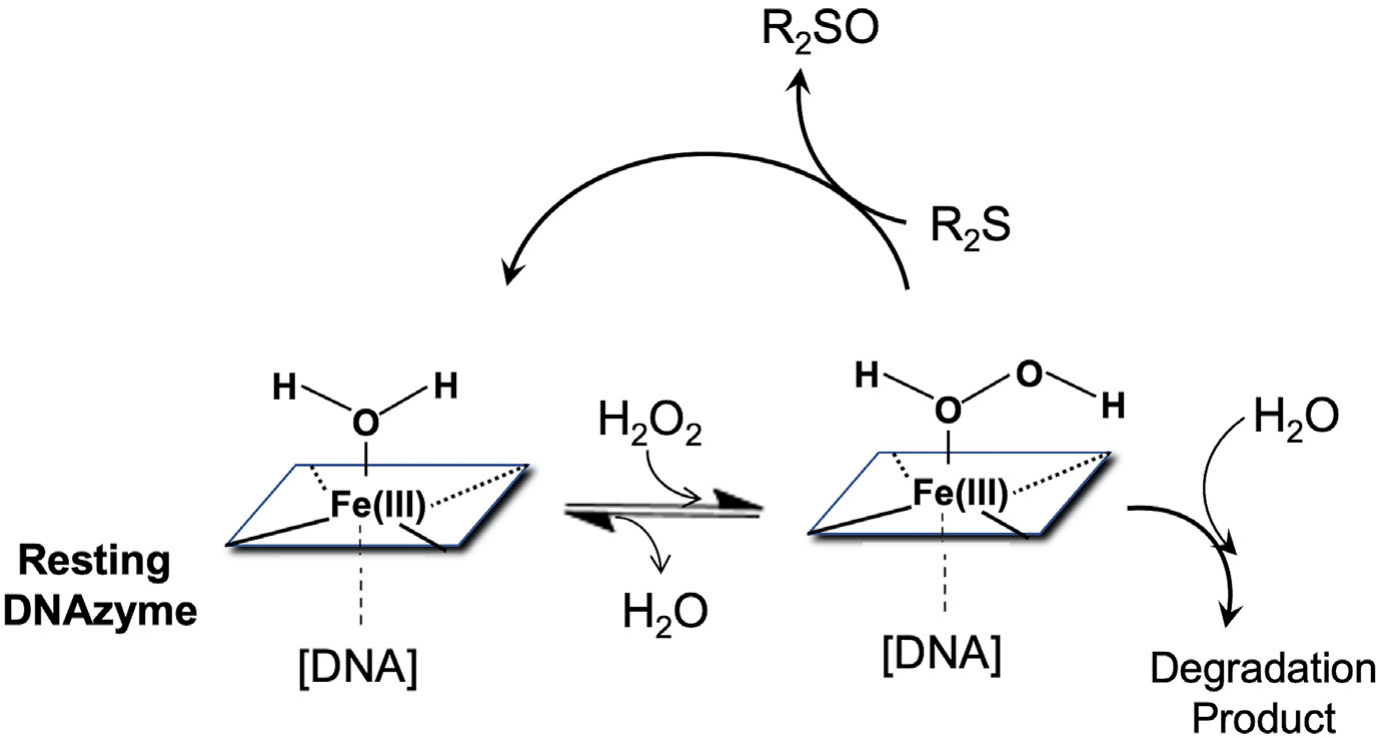
Proposed reaction scheme for the heme•DNAzyme's oxidation of DBT (R_2_S) to DBTO (R_2_SO).

The activated heme species most broadly associated with a variety of oxidative hemoproteins, including peroxidases, metmyoglobin, and cytochromes P450, is Compound I, a heme that is formally two oxidation levels above the resting ferric heme. An enzyme like HRP, whose main activity is 1e^–^ oxidations and a few 2e^–^ oxidation (oxygen atom transfer) reactions, rapidly forms Compound I. Initially, H_2_O_2_ binds to the distal axial site, while in ordinarily 6-coordinate hemoproteins, such as metmyoglobin, there is replacement of the axially bound water to generate an initial Fe(III)heme–H_2_O_2_ complex. Such an initial complex is then able to deprotonate within the enzyme's active site to generate Compound 0 (Figure 9). Compound 0 can then undergo heterolytic cleavage of its O–O bond to form Compound I. In HRP, a Compound I carries out two successive 1e^–^ oxidations, first yielding a Compound II, then ultimately the resting ferric state.

While Compound I is the strong oxidant that is implicated in most of the oxidation reactions carried out by heme enzymes, it has also been noted by different investigators that species that are distinct from a Compound I, such as the heme iron peroxo complex; hydroperoxide complex (Compound 0); or even the initial Fe(III)heme–H_2_O_2_ complex (where the iron moiety provides Lewis Acid activation to the axially bound H_2_O_2_) are capable of catalyzing reactions such as substrate nitration, C–C bond scission, alkene epoxidation, and thioether oxidation (sulfoxidation) (15,22–24).

In this work, the deconvolved UV–vis spectrum of the activated heme species implicated in DBT sulfoxidation (species C•G4) is almost indistinguishable from the starting aquo-species of the heme•DNAzyme (heme•G4) (Figure 7), even when the experiments were performed at 4°C (Supplementary Figure S10). This is a novel result, given that the usual activated heme species such as Compounds 0, I and II, all show distinctive spectral features with respect to that of the resting aquo-Fe(III) state (see above). As illustrated in Figure 9, in the heme•DNAzme (which is spectroscopically similar to hemoproteins, such as metmyoglobin, with 6-coordinate resting states), the first chemical step needs to be a reversible water–H_2_O_2_ exchange at the distal axial coordination site of the enzymatic heme. Given our finding of the almost perfect spectroscopic identity of the C•G4 complex with the heme•G4 complex, we propose that C•G4 does in fact correspond to the hydrogen peroxide complex with the heme•DNAzyme, i.e. the Fe(III)heme-H_2_O_2_ species.

The key question that is raised by the above is whether a Fe(III)heme–H_2_O_2_ complex is a sufficiently powerful oxidant to carry out the oxidation of DBT to DBTO. An important study that corroborates the above hypothesis is by Shaik and coworkers (22), who report a computational investigation of several iron-porphine complexes, including the Fe(III)heme–H_2_O_2_ complex. They find that a Fe(III)heme–H_2_O_2_ complex is, in fact, an efficient oxidant for thioether sulfoxidation, performing that reaction faster even than Compound I (and much more efficiently than Compound 0, which is reported to be a poor sulfoxidation catalyst) (22). Indeed, Shaik and coworkers conclude that the underlying mechanistic underpinning of at least the sulfoxidation reaction catalyzed by Fe(III)heme–H_2_O_2_ is the existence of a low energy barrier for nucleophilic attack by the thioether sulfur on the oxygen of the H_2_O_2_ bound to the distal site of the heme iron. This leads to a proton-coupled heterolytic cleavage of the O–O bond. This key conclusion is indeed consistent with our own finding that the quantitative source of the transferred O atom in our DBTO product is H_2_O_2_; which also indicates, from the high percentage product with ^18^O, that the oxygen transfer likely happens in one step.

The sulfoxidation chemistry of Fe(III)heme–H_2_O_2_ does not apply solely to thiolate-ligated hemes. Shaik and coworkers carried out their computations on iron-porphines with thiol and chloro- as proximal ligands as well as lacking a proximal axial ligand altogether. In all three cases, the Fe(III)porphine–H_2_O_2_ complex was a superior oxidant for thioether substrates (22). This is important in the context of this present work because the heme iron in heme•DNAzymes have been shown to feature a water molecule as the proximal axial ligand. Consistent with predictions from (22), the heme•DNAzyme has a sextet ground state and the deconvolved spectra of C•G4 show spectral features consistent with high spin Fe(III) (i.e., optical bands extending to energies lower than 600 nm). Consequently, the oxo transfer reaction can take place without change of spin to S = ^1^/_2_, as is proposed to occur in thiolate ligated heme (18).

The above mechanistic proposal does not preclude the generation of a Compound I-like activated state in heme•DNAzymes; however, we do not find one to be relevant—at least to thioether oxidation by heme•DNAzymes. For other varieties of oxidation, for instance, Travascio *et al.* (1,51) found that the presence of nitrogenous bases in the reaction buffer solution, particularly sterically hindered bases such as collidine, enhanced the oxidative kinetics of heme•DNAzymes by means of general acid-base catalysis (51). Further, a stopped-flow kinetics and EPR study carried out on two heme•DNAzymes structurally divergent from ours [*inter*molecular GQs: (TTAGGG)_4_ and (TTAGGGA)_4_], under very distinct EPR conditions from our own (in 50 mM potassium phosphate buffer, pH 6.8 with *15* mM H_2_O_2_) reported a small EPR signal for Compound I (52). It also has been shown by a number of other researchers that either ATP or spermine added as an extrinsic general base (53–56) or the strategic positioning of adenines or cytosines, capable of functioning as general bases, at either end or indeed, in the loops of an intramolecular G-quadruplex, lead to enhancement of the kinetics of peroxidation reactions of heme•DNAzymes (57–60). A particularly interesting innovation has linked substrate-specific aptamers with heme•DNAzymes to generate substrate-specific oxidizing DNAzymes (61).

## CONCLUSIONS

In the case of oxidation of thioethers to sulfoxides by the heme•DNAzyme, we present experimental evidence that an Fe(III)heme–H_2_O_2_ complex functions as the active heme species for sulfoxdation reactions. We see changes in the EPR that are consistent with an S = ^1^/_2_ intermediate. The extracted UV–vis spectra also suggest an S = ^1^/_2_ six coordinate heme. The only intermediate that builds up is an S = ^1^/_2_ heme, that we propose to be the H_2_O_2_–heme•G4 complex. The above does not preclude Compound I as a possible oxidant; however, if it is playing a role, it does not build up. In summary, we present spectroscopic evidence that a heme-H_2_O_2_ initial adduct—which has been proposed independently to be a powerful oxidant for thioether oxidation—is responsible for this particular class of oxidation reactions catalyzed by heme•DNAzyme systems.

## Supplementary Material

gkab007_Supplemental_File
