# Supplementary material for: A heme•DNAzyme activated by hydrogen peroxide catalytically oxidizes thioethers by direct oxygen atom transfer rather than by a Compound I-like intermediate
Source: Nucleic Acids Res. 2021 Jan 21;49(4):1803–15. doi: 10.1093/nar/gkab007 (PMC7913675; doi:10.1093/nar/gkab007)
Supplement: gkab007_Supplemental_File [file gkab007_supplemental_file.pdf]

A Heme•DNAzyme Activated by Hydrogen Peroxide Catalytically Oxidizes Thioethers by Direct Oxygen Atom Transfer rather than by a Compound I-like Intermediate

Nisreen M. Shumayrikh[1], Jeffrey J. Warren[1], Andrew J. Bennet[1], and Dipankar Sen[1, 2]*

[1]Department of Chemistry, Simon Fraser University,

Burnaby, British Columbia V5A 1S6, Canada

[2]Department of Molecular Biology & Biochemistry, Simon Fraser University,

Burnaby, British Columbia V5A 1S6, Canada

*Email: sen@sfu.ca

Supporting Information

Description of the software and treatment of the kinetic data

The experimental traces recorded from 300 nm to 740 nm were exported as glb file extensions and processed by Pro-KIV Global Analysis Software. Singular value decomposition (SVD) was performed, by which the data matrix was decomposed into 3 matrices with the form of: $Y= U . S . V^T$

Each column of the U matrix represents the time evolution of the reaction. U is a matrix of column vectors where each column is an eigenvector in the time domain. V^T is a matrix of column vectors where each column is an eigenvector in the wavelength domain. S is a diagonal matrix with the singular values S. Each element of S represents the contribution of the corresponding basis spectra to the observed data.

SVD analysis can be viewed as a reduced representation of the data matrix as an ordered set of basis spectra and corresponding time-dependent amplitudes as well as their singular values in descending order. It should be noted that SVD output is completely abstract and has no physical or chemical meaning (abstract factor analysis). However, the SVD application aids the analysis in speeding up the subsequent numerical calculations and reduce noise in the dataset. The SVD output is then analyzed by global optimization of the provided reaction parameters using the Marquardt-Levenberg algorithm (Reference S1). The reaction scheme is input into the Analysis Equations window, as successive steps. Compiling the model generates the rate parameters associated with each step of the entered scheme. The spectrum of the starting material (the ferric DNAzyme), as well as of the DBT and the DBTO, were collected under the same experimental conditions from 1000 spectra each. These 1000 spectra were averaged, and saved as a single spectrum, then fed into the software as known spectra. Hydrogen peroxide was included in the reaction scheme but not incorporated into the regression analysis and was assigned as a colourless species. In order to run the analysis, the concentrations of each of the starting materials (heme/G4-DNAzyme, H_2O_2, and DBT) were entered, with an initial estimate given for each rate constant. Initial guesses for rate constants were input based on kinetics runs with and without substrate. Residual plots were analysed to ensure that kinetics phases were not systematically excluded from the models. Computed component spectra had no negative absorbance features and calculated extinction coefficients were of intensities consistent with other heme complexes reported in the literature (References S2-S6). The calculated rate constants obtained from the software analysis were reported as the mean attained from three replicate experiments with their standard deviation errors. Figure S1 shows the order of steps by which the analysis process is accomplished.

In this work, a number of models as well as values of rate parameters have been tested in order to achieve reasonable calculated spectra and residual plots. Also, to test the robustness of our hypothesized model, one individual step was systematically removed from the scheme, followed by re-performing the analysis and inspection of the residual plots (see Figure S10)

REFERENCES

1. Kanzow, C., Yamashita, N., Fukushima, M. (2004) Levenberg–Marquardt methods with strong local convergence properties for solving nonlinear equations with convex constraints. *J. Comput. Appl. Math.* **172**, 375-397.
2. Henry, E. R., Hofrichter, J. (1992) Singular Value Decomposition: Application to Analysis of Experimental Data. *Meth. Enzymol.* **210**, 129.
3. Maeder, M. & Zuberbuehler, A. D. (1990) Nonlinear least-squares fitting of multivariate absorption data. *Anal. Chem.* **62**, 2220–2224.
4. Van Niekerk, W., Pienaar, J. J., Lachmann, G., Van Eldik, R. & Hamza, M. A (2007) Kinetic and mechanistic study of the chromium (VI) reduction by hydrogen peroxide in acidic aqueous solutions. *Water S. A.* **33**, 619–625.
5. Melvin, W. S., Rablen, D. P. & Gordon, G. (1972) Kinetics of substitution reactions of poly(ethylenamine)nickel(II) complexes. *Inorg. Chem.* **11**, 488–493.
6. Groves, J. T., Lee, J. & Marla, S. S. (1997) Detection and Characterization of an Oxomanganese (V) Porphyrin Complex by Rapid-Mixing Stopped-Flow Spectrophotometry. *Science* **7863**, 6269–6273.

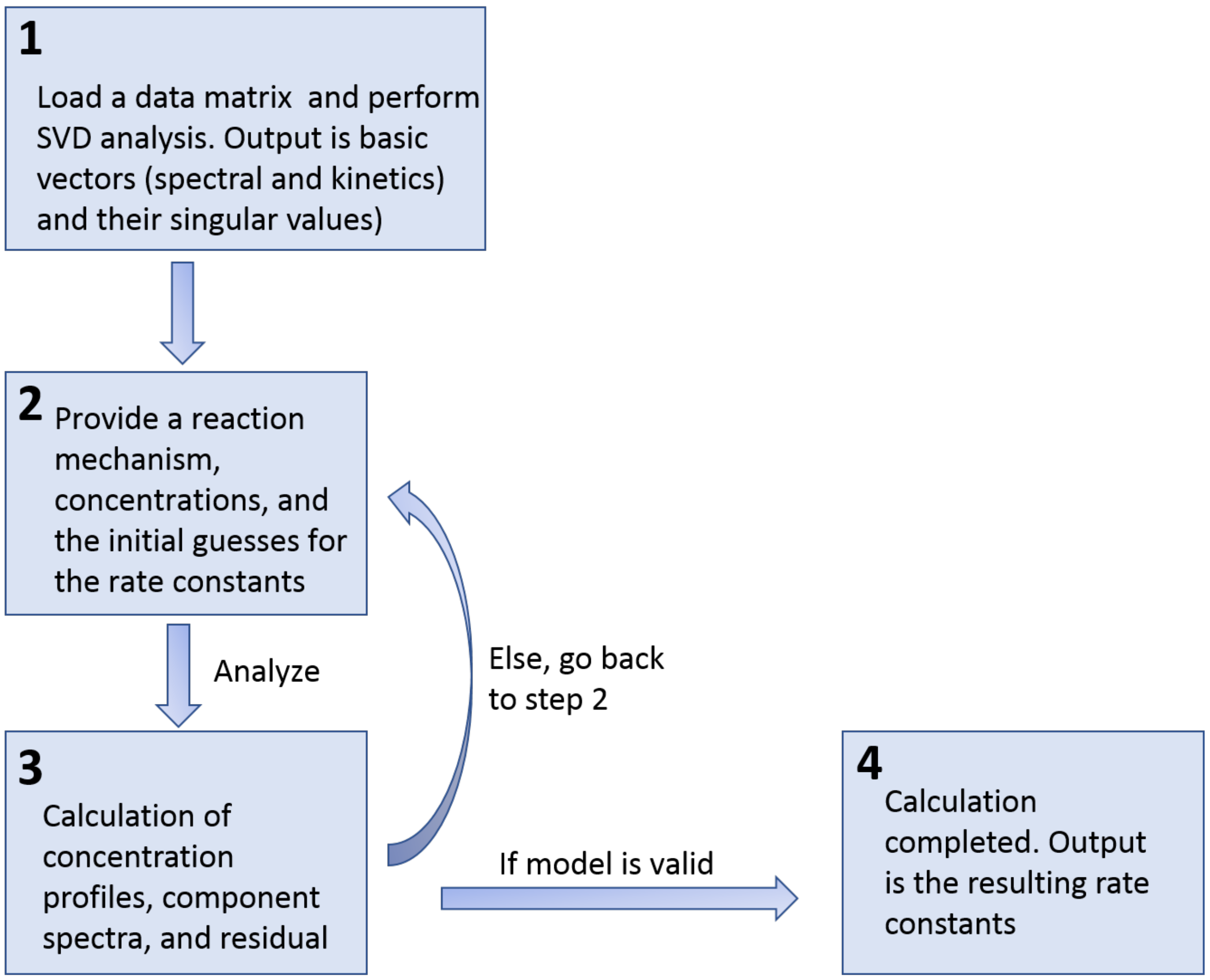

Figure S1: Schematic representation of steps flow during the fitting process by Pro-KIV Software.

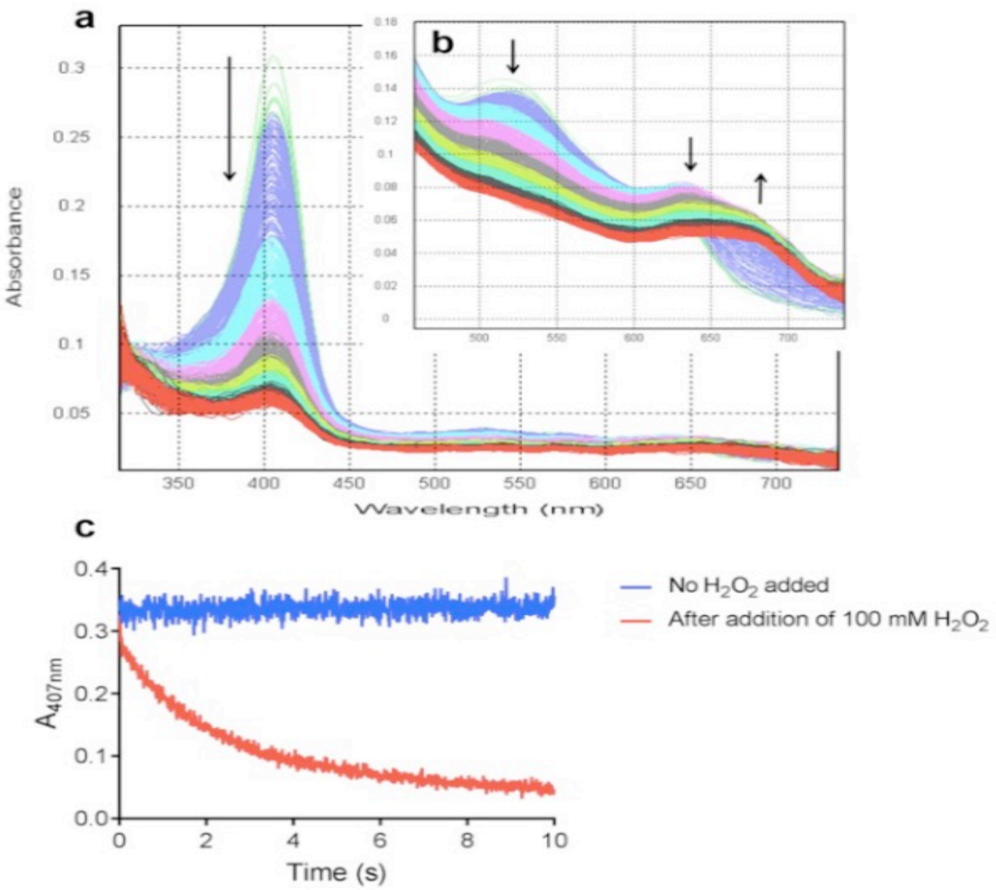

Figure S2: Spectral change induced in the reaction of heme/G4-DNAzyme with 100 mM H_2O_2 in absence of substrate at pH 8.0, 21°C followed over 10 seconds. (***a***) Soret region, (***b***) visible region, and (***c***) graph of the change in absorbance at the Soret wavelength (407 nm). 7 µM (***a***) or 15 µM (***b***) of heme/G4-DNAzyme was used for the measurements on a stopped-flow rapid scan system.

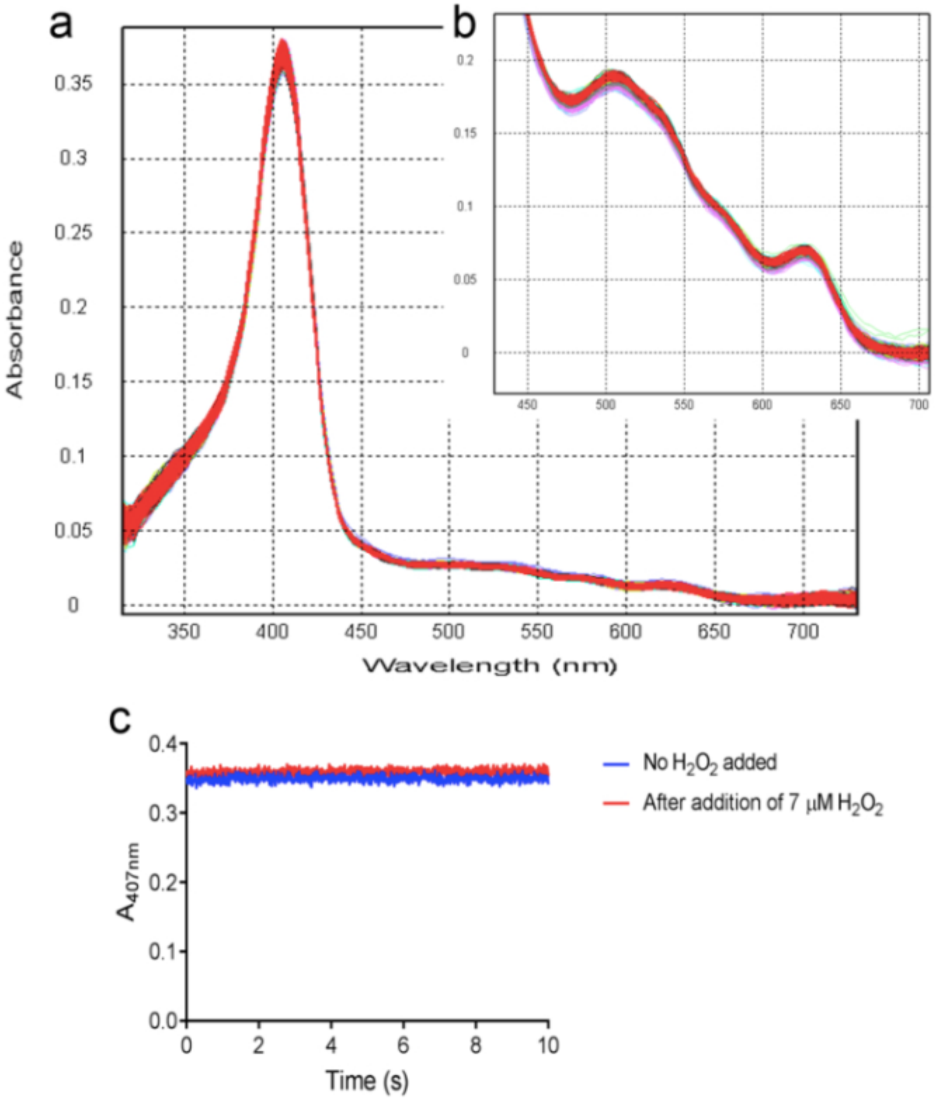

Figure S3: Spectral change induced in the reaction of heme/G4-DNAzyme with 7 μM H_2O_2 in absence of substrate at pH 8.0, 21 °C, followed over 10 seconds. (***a***) Soret region, (***b***) visible region, and (***c***) graph of the change in absorbance at the Soret wavelength (407 nm). 7 μM (***a***) or 15 μM (***b***) of heme/G4-DNAzyme was used for the measurements on a stopped-flow rapid scan system.

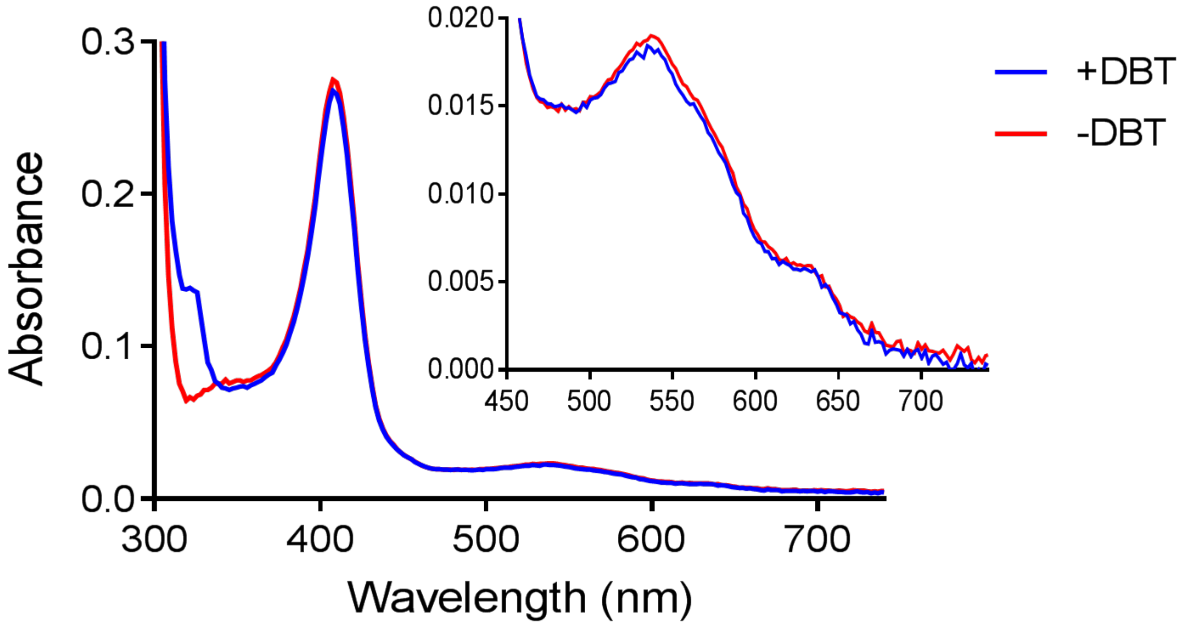

Figure S4: The spectrum of Ferric(III) heme bound to G4 DNA (the Fe(III)heme•DNAzyme) in the presence (blue trace) and absence (red trace) of substrate**.** Scans were taken in reaction buffer [40 mM HEPES-NH_4OH, pH 8.0, 20 mM KCl, 1% DMF, 0.05% Triton 100-X containing 25% methanol].

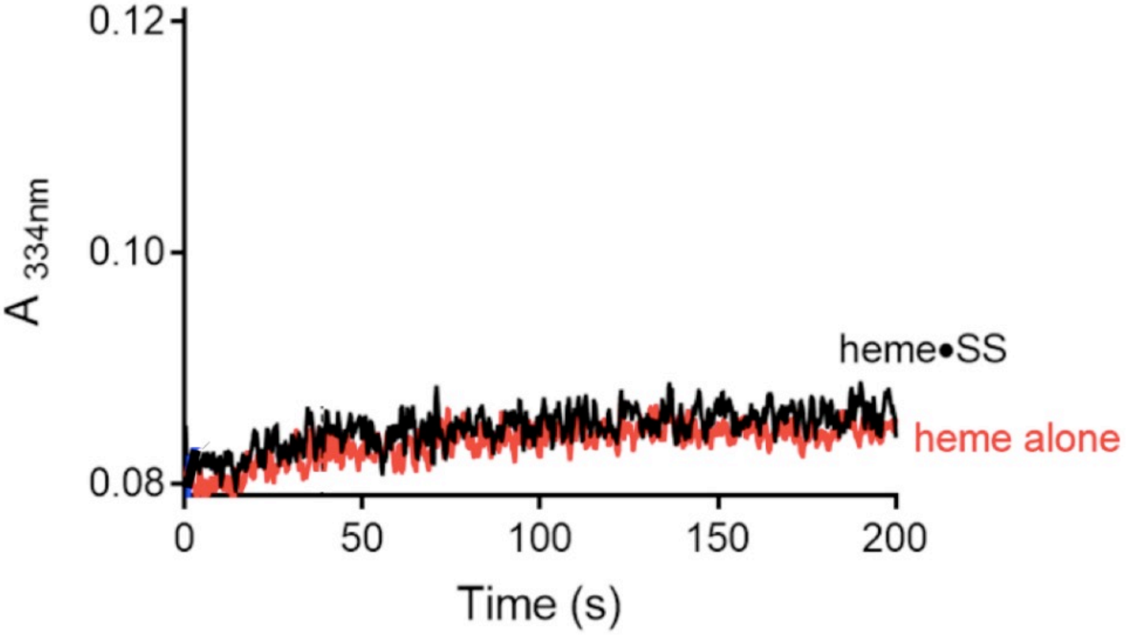

Figure S5: Plots of the change in absorbance at 334 nm over 200 s time in the uncatalyzed reaction of heme•SS (black trace) and the uncatalyzed reactions of heme alone, in the absence of any added DNA (red trace).

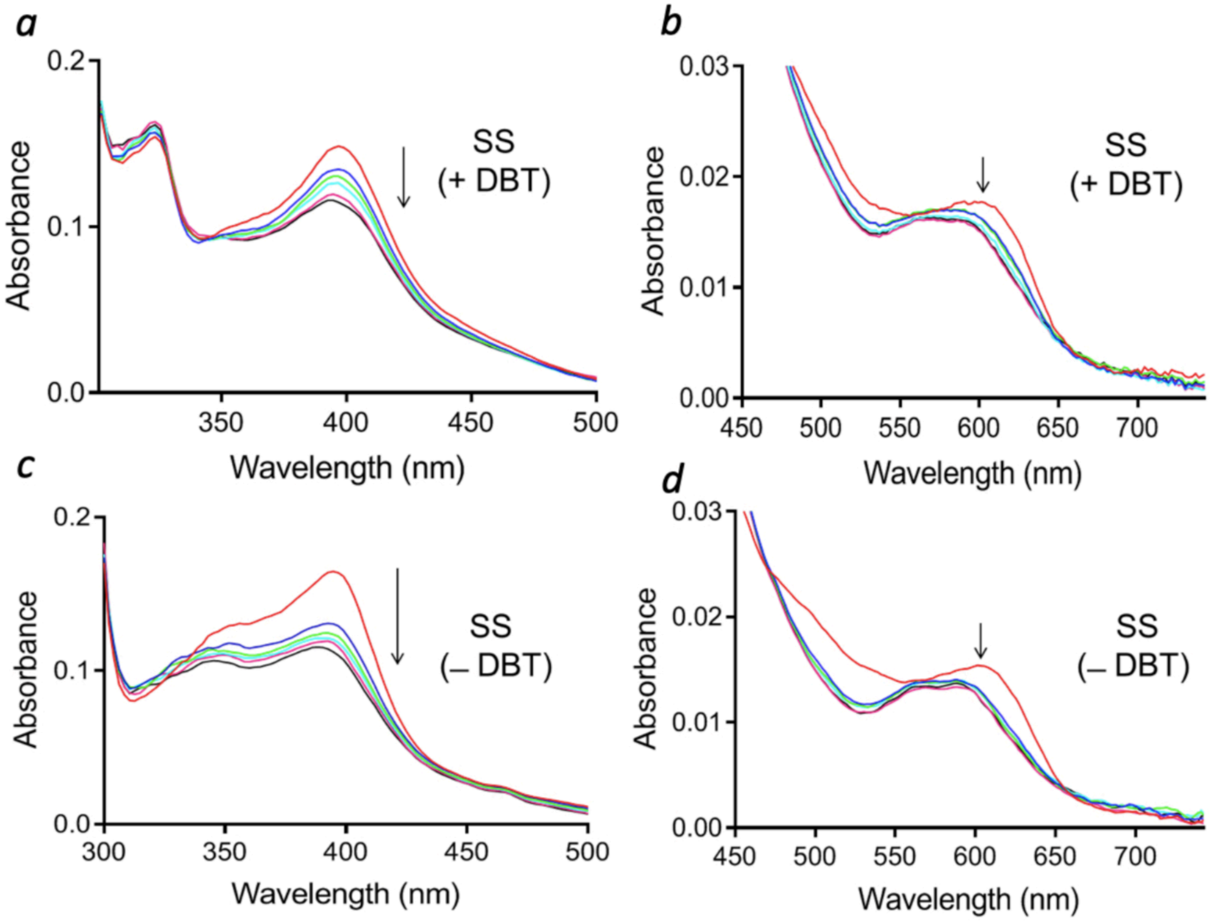

Figure S6: Time-dependence of spectral changes that are associated with all the species present during the reaction time course. Shown are hemin in the presence of the non-interacting SS DNA, in the presence (***a, b***) and the absence (***c, d***) of added DBT substrate. t = 0 s traces are shown in red and t = 200 s in black.

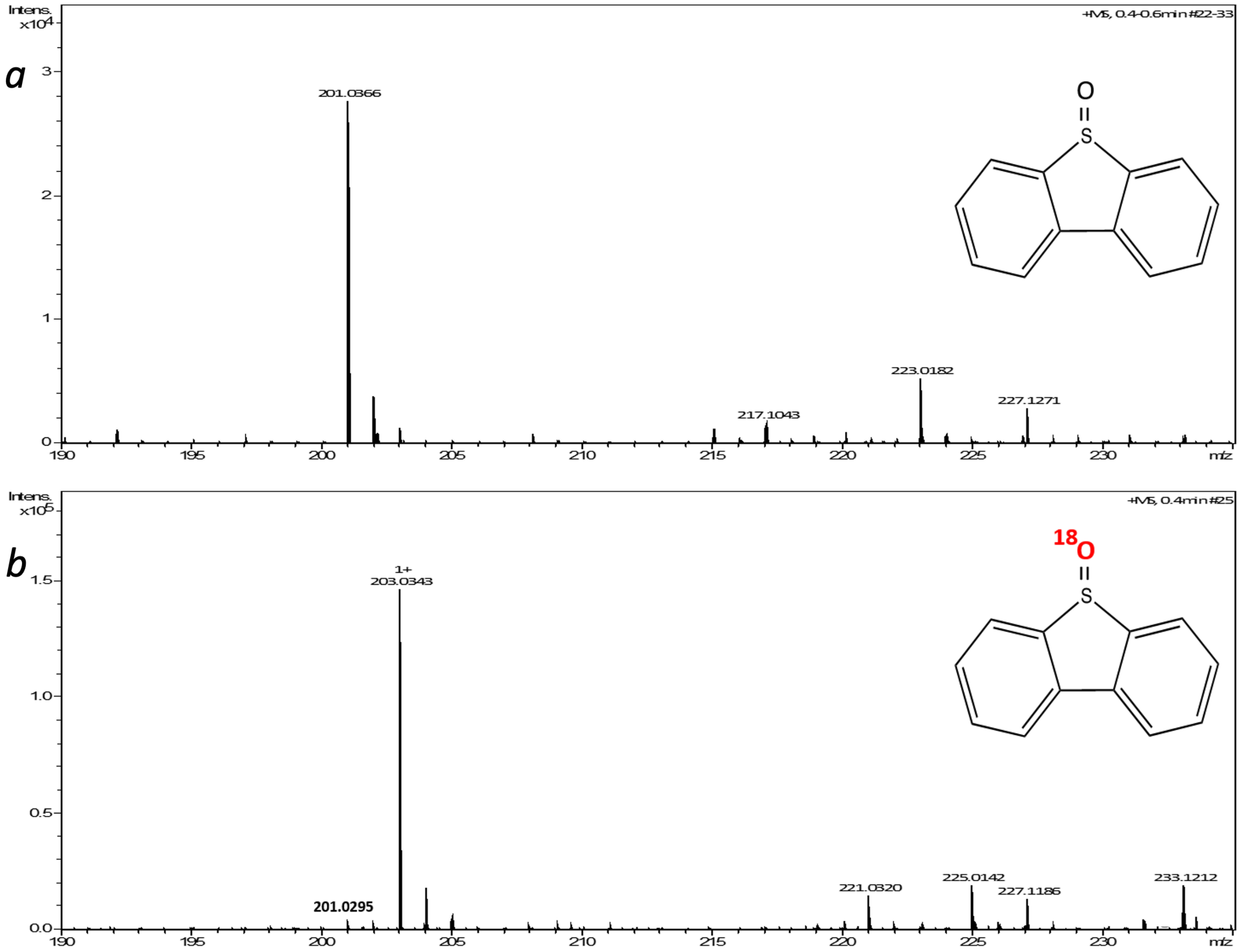

Figure S7: ESI mass spectra showing that the source of the oxygen atom in the DBTO product is exclusively from H_2O_2. (***a***) is a control showing the mass fragmentation pattern for DBTO generated by hemin•DNazyme catalyzed reaction using H_2O_2. Adducts (+ H^+) with m/z of 201.036 and (+ Na^+) with m/z of 223.018 can be seen. (***b***) is the mass fragmentation pattern for $DBT^{18}O$ generated by hemin•DNazyme catalyzed reaction using $H_2{}^{18}O_2$. Adducts (+ H^+) with m/z of 203.034, 201.029 and (+ Na^+) with m/z of 225.014 can be seen.

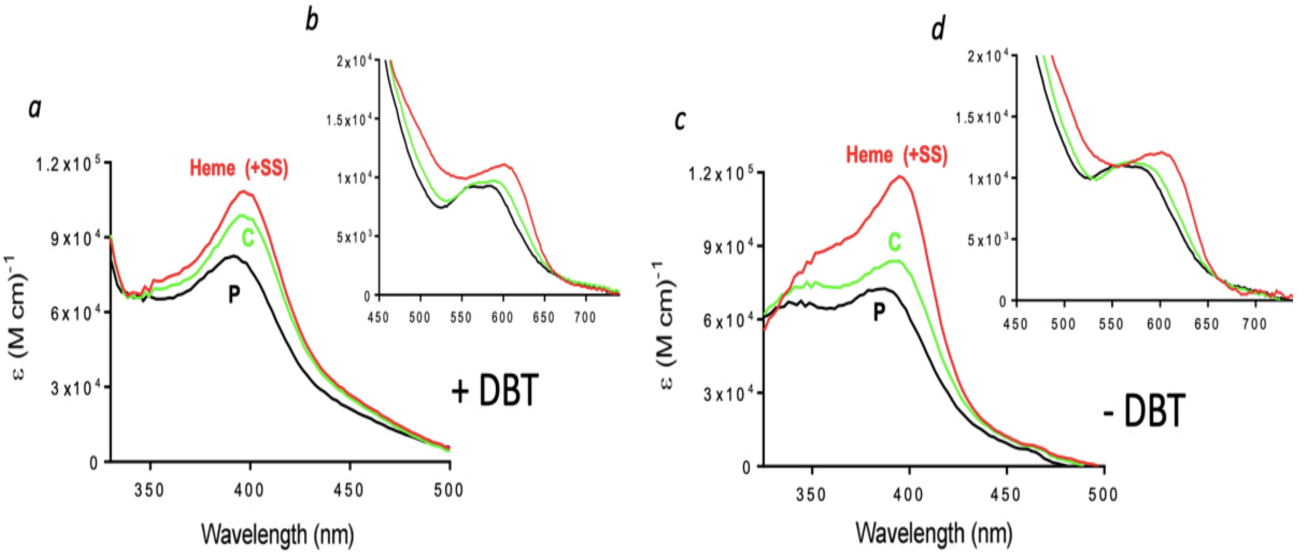

Figure S8: Deconvolved spectra of the hemin in the presence of the non-interacting DNA, SS, in the presence of the DBT substrate (***a, b***); and in the absence of the DBT substrate (***c, d***).

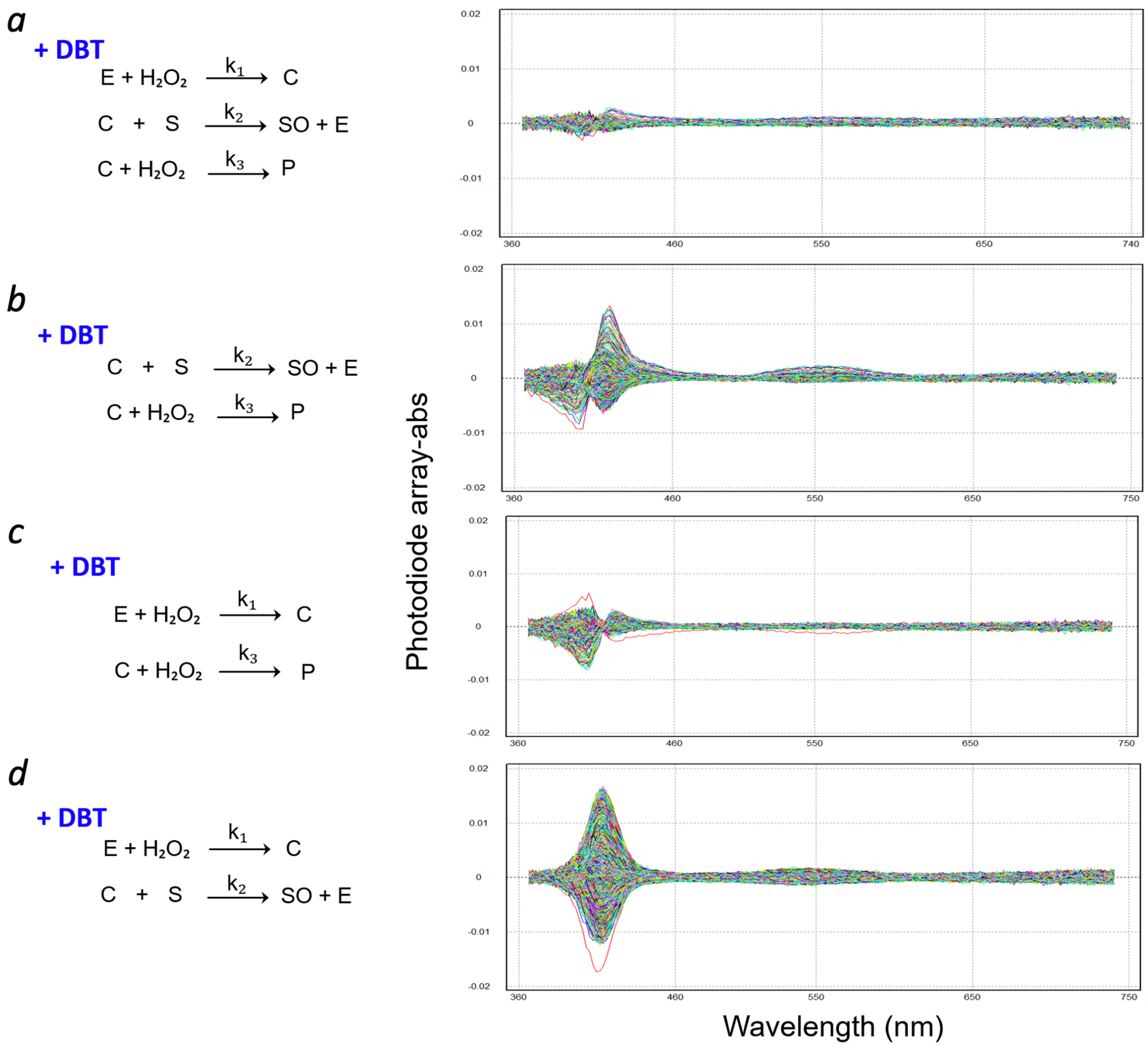

a
+ DBT
E + H2O2 k1 → C
C + S k2 → SO + E
C + H2O2 k3 → P
b
+ DBT
C + S k2 → SO + E
C + H2O2 k3 → P
c
+ DBT
E + H2O2 k1 → C
C + H2O2 k3 → P
d
+ DBT
E + H2O2 k1 → C
C + S k2 → SO + E
Photodiode array-abs
Wavelength (nm)
0.02
0.01
0
-0.01
-0.02
360
460
550
650
740
750

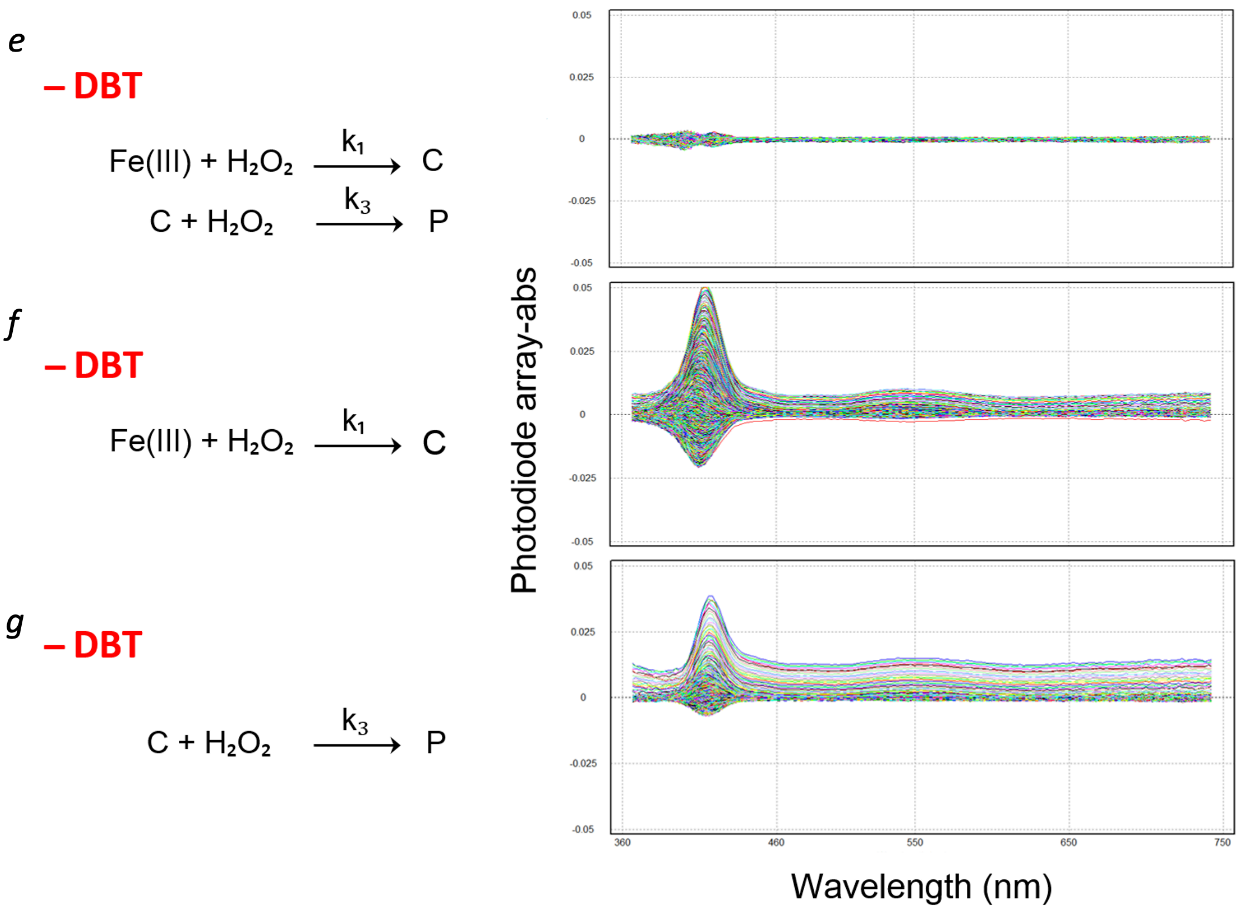

Figure S9: Residual plots are shown as a function of wavelength. Panels ***a-d*** represent residual plots from + DBT and panels ***e-g*** represent -DBT data sets, respectively. The complete model and the ones with the omitted step are shown next to the plots. In these models, E is the hemin•DNAzyme, C is the activated species, S is the substrate, SO is the oxidized product, P is the disaggregated product. For consistency, all residual plots are shown with the same x and y scale.

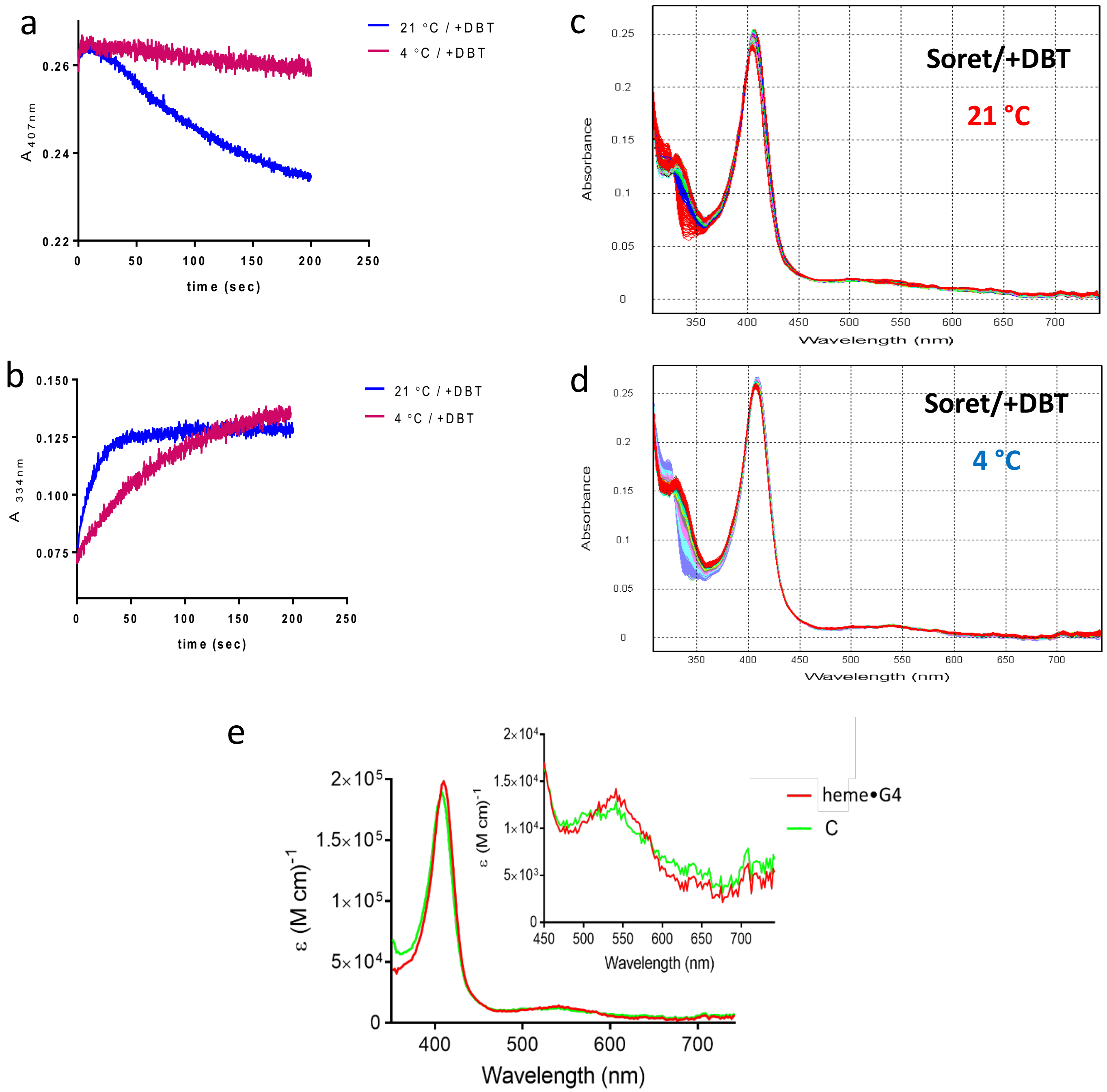

Figure S10: A comparison of stopped flow experiments in presence of DBT performed at 21° C and 4 ºC. (***a***) and (***b***) shows the change in the absorbance at 407 nm (Soret) and 334 nm (DBTO) over time respectively. (***c***) and (***d***) shows the collected raw data by PDA at 21° C and 4° C respectively. (***e***) shows the extracted component spectra of E (heme•G4) and C (activated species) after fitting the 4° C data. The fitting was performed using the same model used for the 21° C data except without k_3—which can be removed owing to its minor contribution at 4° C.

Calculated rate constants at 4 ºC are: $k_1 = (5.6 \pm 0.1) \times 10^2\ M^{-1}s^{-1}$; $k_{-1} = (4.6 \pm 0.2) \times 10^3\ s^{-1}$; and $k_2 = (1.1 \pm 0.1) \times 10^2\ M^{-1}s^{-1}$.

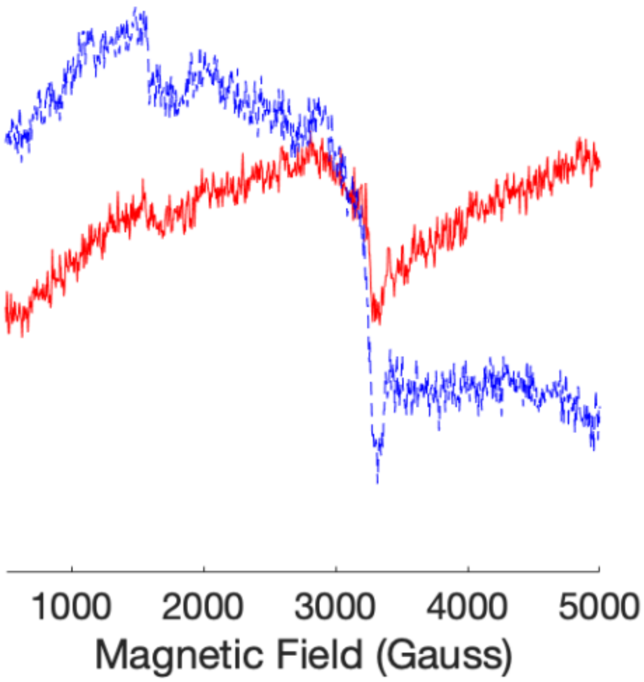

Figure S11: EPR spectra of G4•heme (without H_2O_2) (blue spectrum) and G4•heme + H_2O_2 (red spectrum) after annealing at room temperature for 2 minutes. Concentrations are 7 µM heme, 50 µM DNA, and 500 µM H_2O_2.
